# The role of autoproteolysis and mitoribosomal proteins in the regulation of the mitochondrial LACTB tumor suppressor

**DOI:** 10.1038/s44319-026-00936-9

**Published:** 2026-09-26

**Authors:** Sara Escudeiro-Lopes, Juan M Gonzalez-Morena, Alzbeta Baudysova, Michal Svoboda, Pavel Marasek, Gabriel Rodriguez-Gomez, Jessica Ferreira Mendes, Chung Weng Phang, Sravan Kumar Miryala, Pavel Jakoube, Beata Kovarova, Susana Machado, Yuxiong Feng, Zuzana Keckesova

**Affiliations:** 1 Institute of Organic Chemistry and Biochemistry, Czech Academy of Sciences, Prague, Czech Republic; 2 Faculty of Science, Charles University, Albertov 6, 128 43 Prague, Czech Republic; 3 Zhejiang Provincial Key Laboratory of Pancreatic Disease, First Affiliated Hospital, and Institute of Translational Medicine, Zhejiang University School of Medicine, Hangzhou, China; 4 Cancer Center, Zhejiang University, Hangzhou, China

**Keywords:** Cancer, Post-translational Modifications & Proteolysis, Signal Transduction

## Abstract

Tumor suppressors represent one of the first lines of defense against malignant transformation, and their inactivation in cells leads to the onset of tumorigenesis. Cancer cells employ various mechanisms to inactivate cellular tumor suppressors, including epigenetic silencing and mutagenesis. Less understood are the mechanisms by which cancer cells inactivate tumor suppressors post-translationally. Here, we uncovered a previously undescribed post-translational mechanism by which cancer cells inactivate the potent mitochondrial tumor suppressor LACTB in breast cancers. We discovered that the substrate of LACTB can be LACTB itself; LACTB possesses autoproteolytic activity, which is important for modulating its tumor suppressor activity. We demonstrate that cancer cells exploit this feature of LACTB to induce LACTB’s self-degradation. This is mechanistically realized through upregulation of mitochondrial MRPS34 protein, which, through interaction with LACTB, is a positive regulator of LACTB’s autoproteolytic activity and a negative regulator of LACTB. This study provides insights into how cancer cells fine-tune the expression and activity of tumor suppressors to promote tumorigenesis.

## Synopsis

In breast cancer cells, the mitochondrial tumor suppressor LACTB is repressed through enhancement of its autoproteolytic activity, resulting in self-degradation. The mitochondrial small ribosomal subunit MRPS34 is upregulated to interact with  LACTB, activating auto-proteolysis.

- LACTB possesses autoproteolytic activity.
- MRPS34 is a negative regulator of LACTB.
- MRPS34 enhances the autoproteolytic activity of LACTB.

## Abstract (web summary)

In breast cancer cells, the mitochondrial tumor suppressor LACTB is repressed through enhancement of its autoproteolytic activity, resulting in self-degradation. The mitochondrial small ribosomal subunit MRPS34 is upregulated to interact with  LACTB, activating auto-proteolysis.

## Graphical abstract

**Figure:**
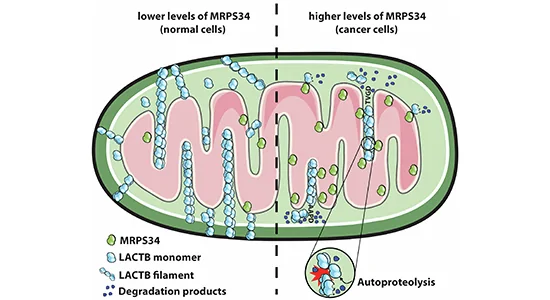

## Introduction

Deactivation of tumor suppressors is a crucial step in the development and progression of malignancies. In human cells, this can occur through the complete loss/inactivation of a particular tumor suppressor, via cellular processes such as gene mutation (Stratton et al, 2009; Greenman et al, 2007), aberrant splicing (Yang et al, 2019; Sciarrillo et al, 2020), and epigenetic silencing (Esteller, 2011; Baylin and Jones, 2016). The complete loss of a tumor suppressor is described by Knudson’s two-hit hypothesis, which stipulates that inactivation of both alleles of a tumor suppressor gene is required to promote tumor progression (Tomlinson et al, 2002). However, subsequent research revealed the existence of a different class of tumor suppressors termed “haploinsufficient/dose-dependent”, where loss of only one allele of such a tumor suppressor already contributes to the tumorigenic process, which is sometimes manifested through lower expression levels of such proteins (Santarosa and Ashworth, 2004; Inoue and Fry, 2017). Examples of haploinsufficient tumor suppressors include p27Kip1, NF1, and PTEN, among others (Inoue and Fry, 2017). These tumor suppressors represent attractive therapeutic targets for novel cancer treatments, since, in many cases, they are never completely absent from the cell. Recently, beta-lactamase-like protein (LACTB) was also characterized as a novel haploinsufficient mitochondrial tumor suppressor in breast cancer cells (Keckesova et al, 2017).

LACTB is a ubiquitously expressed mitochondrial protein, derived from the bacterial penicillin-binding/β-lactamase proteins (PBP-βL) family, which eukaryotic cells acquired during endosymbiosis (Smith et al, 2001; Peitsaro et al, 2008). Eukaryotic LACTB shares conserved active site motifs with bacterial PBP-βL proteins, including the catalytic serine site -SXXK- (Peitsaro et al, 2008). In bacteria, PBP-βL proteins are involved in peptidoglycan synthesis, which is important for the bacterial cell wall biogenesis (Ghuysen, 1997; Meroueh et al, 2003). Since eukaryotic cells lack bacterial peptidoglycans, the need for LACTB’s catalytic activity is currently unknown.

LACTB was first described as a tumor suppressor in breast cancer (Keckesova et al, 2017) and follow-up studies extended its tumor suppressor role to other cancer types, such as colorectal cancer (Zeng et al, 2018; Zhou et al, 2022; Xu et al, 2020; Wang et al, 2020), hepatocellular carcinoma (Xue et al, 2018), glioma (Li et al, 2019), gastric cancer (Yang et al, 2021), melanoma (Ma et al, 2021; Liu et al, 2021), lung cancer (Xu et al, 2022) and ovary cancer (Cutano et al, 2022). However, studies in pancreatic cancer (Xie et al, 2021) and in nasopharyngeal carcinoma (Peng et al, 2021) showed that higher expression of LACTB was associated with cancer progression, thus identifying LACTB as a possible tumor promoter in these cancer types. LACTB’s tumor suppressor function is realized through modulation of lipid metabolism, inhibition of cellular proliferation and epithelial-mesenchymal transition (EMT), and induction of differentiation and cancer cell death, through several different mechanisms of action in a tissue-specific manner (Keckesova et al, 2017; Xue et al, 2018; Li et al, 2019; Ma et al, 2021; Gonzalez-Morena et al, 2023; Zeng et al, 2018). Collectively, these studies showed that downregulation/inactivation of LACTB in cancer cells, which occurs through both transcriptional and post-translational mechanisms, is a common mechanism by which cancer cells counteract LACTB’s negative effects on their viability. At the transcriptional level, this regulation occurs through different mechanisms: LACTB silencing via microRNA binding to its 3’-UTR, with microRNA-1276 in colon cancer cells (Wang et al, 2020) and MiR-125b-5p in non-tumorigenic THP-1 macrophages (Lu et al, 2016). In colorectal cancer, LACTB downregulation is associated with its promoter methylation and histone H3 hypoacetylation, showing the involvement of epigenetic modifications in LACTB repression (Zeng et al, 2018). In melanoma, the transcription factor SOX10 binds to the LACTB promoter and negatively regulates its transcription (Ma et al, 2021). However, although some information is available on the transcriptional regulation of LACTB, the mechanisms underlying post-translational downregulation of LACTB by cancer cells remain unknown.

The aim of our work was to uncover this important aspect of post-translational regulation of LACTB in breast cancer cells, which began with our search for the LACTB substrate. Using the adapted Proteomic Identification of Cleavage Sites (PICS) assay, we identified that the LACTB substrate could be LACTB itself and that LACTB possesses autoproteolytic activity. This activity is important for LACTB’s tumor-suppressive ability and targets the AAPD and TVGD amino acid sequences within LACTB. The autoproteolytic activity of LACTB is co-opted by cancer cells to promote efficient downregulation of the tumor suppressor protein. Mechanistically, this process is driven by upregulation of the 28S ribosomal protein S34 (MRPS34), which negatively regulates LACTB by enhancing its autoproteolytic activity. As a result, LACTB undergoes self-degradation in cancer cells, thereby promoting cancer cell proliferation and tumor growth.

## Results

### LACTB possesses autoproteolytic activity

**Figure 1:**
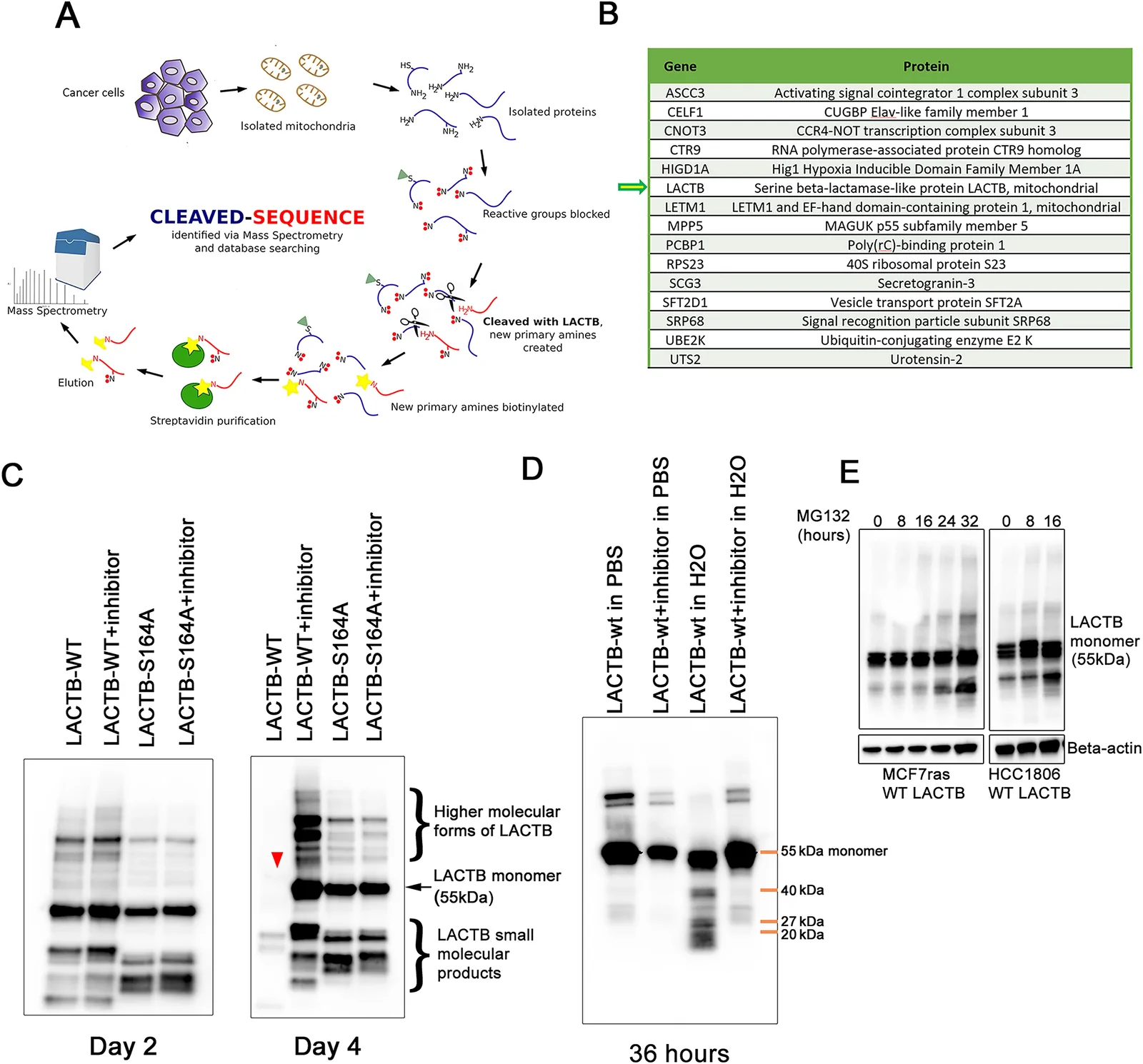
Identification of LACTB substrates. (**A**) Schematic representation of the workflow of the modified PICS assay. Assay was performed with both wt LACTB and S164A LACTB. Arrowhead points to the decrease in the LACTB monomer levels. (**B**) Table summarizing the potential LACTB substrates. (**C**) Recombinant LACTB-WT and S164A LACTB, with and without an inhibitor of its activity (Z-AAD-CMK), were incubated at different time points at 25 °C and visualized by Western blot. The experiment was performed twice (*N* = 2). The red arrow points to the disappearance of the LACTB monomer. (**D**) LACTB-WT, with and without inhibitor, was incubated either in PBS or in H_2_O for 36 h at 25 °C. The experiment was performed three times. (**E**) Western blot analysis of LACTB-WT (induced by doxycycline for 24 h) in MCF7R and HCC1806 cells upon treatment with 20 µM MG132. Membrane was probed with LACTB and beta-actin antibodies. Source data are available online for this figure.

**Figure EV1:**
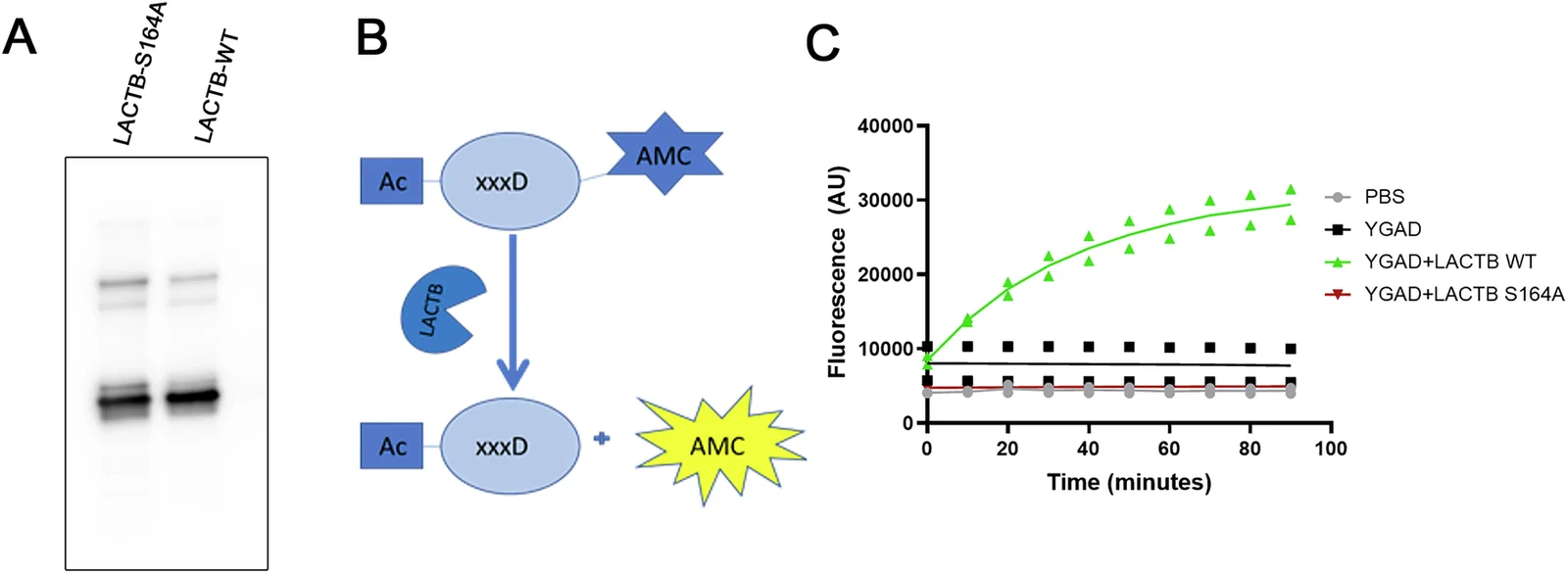
Expression and activity of LACTB recombinant protein. (**A**) The expression level of LACTB-WT and LACTB-S164A recombinant proteins is shown by Western blot analysis. (**B**) Graphical illustration of LACTB’s enzymatic activity assay using tetrapeptides (such as YVAD and YGAD) linked to AMC fluorescent probe. Upon cleavage of the probe by LACTB, the AMC moiety is released and emits fluorescence. (**C**) Comparison of the cleavage ability of LACTB-WT and LACTB-S164A catalytic mutant against the YGAD probe. The experiment was performed twice (*N* = 2). Mean ± SD.

To identify the substrate of LACTB, we set up and optimized a modified protocol of the Proteomic Identification of Cleavage Sites (PICS) assay (Schilling and Overall, 2008) for uncovering substrates of proteases (Fig. 1A). In this approach, we utilized the MCF7ras cell line (MCF7R), whose growth, as shown by us previously, is negatively affected by LACTB-WT induction, but not by the catalytic mutant LACTB, implying the presence of LACTB substrate in this cell line (Keckesova et al, 2017). We isolated the full-length proteome of the mitochondria-enriched fraction from MCF7R cells, followed by blocking reactive groups within the proteome and the addition of wild-type recombinant LACTB. Cleavage of its substrate by LACTB revealed new reactive N-termini within the cleaved substrate, which were biotinylated, captured, and analyzed by mass spectrometry. In parallel, the assay was performed under the same conditions in the presence of a catalytically inactive S164A LACTB mutant as a negative control. The modified assay was performed in two biological replicates (two biological replicates for LACTB-WT and two biological replicates for S164A LACTB). The results identified 15 proteins with a high probability of being the substrate/s of LACTB (Fig. 1B). The recombinant LACTB protein used in the modified PICS assay was isolated from human HEK293T cells and validated by Western blot analysis and by enzymatic activity assay for the correct size, expression, and functional catalytic activity (Fig. EV1A–C).

Once the list of potential LACTB substrates was generated, we were surprised to notice that LACTB-WT was amongst the substrate hits (Fig. 1B; Dataset EV1). Therefore, in this manuscript, we set out to investigate whether LACTB can serve as its own substrate and possess autoproteolytic activity. We incubated recombinant LACTB-WT and LACTB-S164A, with and without the inhibitor of its enzymatic activity (Granzyme B inhibitor—Z-AAD-CMK, (Bachovchin et al, 2014)), for 2 days and 4 days in PBS buffer and examined the resulting stability of LACTB by Western blot (Fig. 1C). The results showed that LACTB-WT was efficiently degraded at 4 days of incubation. This degradation is completely abolished in the presence of the inhibitor of LACTB’s enzymatic activity, where LACTB protein levels did not change at 4 days of incubation. The same result as with the chemical inhibitor of LACTB’s catalytic activity was observed with the S164A LACTB catalytically inactive mutant, whose protein levels were also stable at 4 days of incubation time. These data demonstrate that LACTB-WT indeed possesses autoproteolytic activity mediated by the catalytic serine residue at position 164, and that the observed effects are not attributable to contaminating protease activity. No differences in LACTB-WT stability were observed at an earlier time point (2 days), suggesting that while LACTB is capable of autoproteolytic activity, this activity occurs with slow kinetics. It might require specific cellular conditions, cofactors, or additional stimuli for efficient execution or might be slowed down in the presence of certain environmental conditions. Indeed, LACTB’s autoproteolytic activity manifested at earlier time points (36 h) in H_2_O, as opposed to PBS (Fig. 1D). Next, we examined whether the autoproteolytic ability of LACTB-WT manifests itself also in a physiological cellular context. Upon treatment of MCF7R and HCC1806 cells with the proteasome inhibitor MG132, we observed increased accumulation of LACTB-WT degradation products (Fig. 1E), confirming the ongoing autoproteolytic activity of LACTB in these cancer cell lines.

### The autoproteolytic activity of LACTB is targeting the AAPD/TVGD amino acid residues

**Figure 2:**
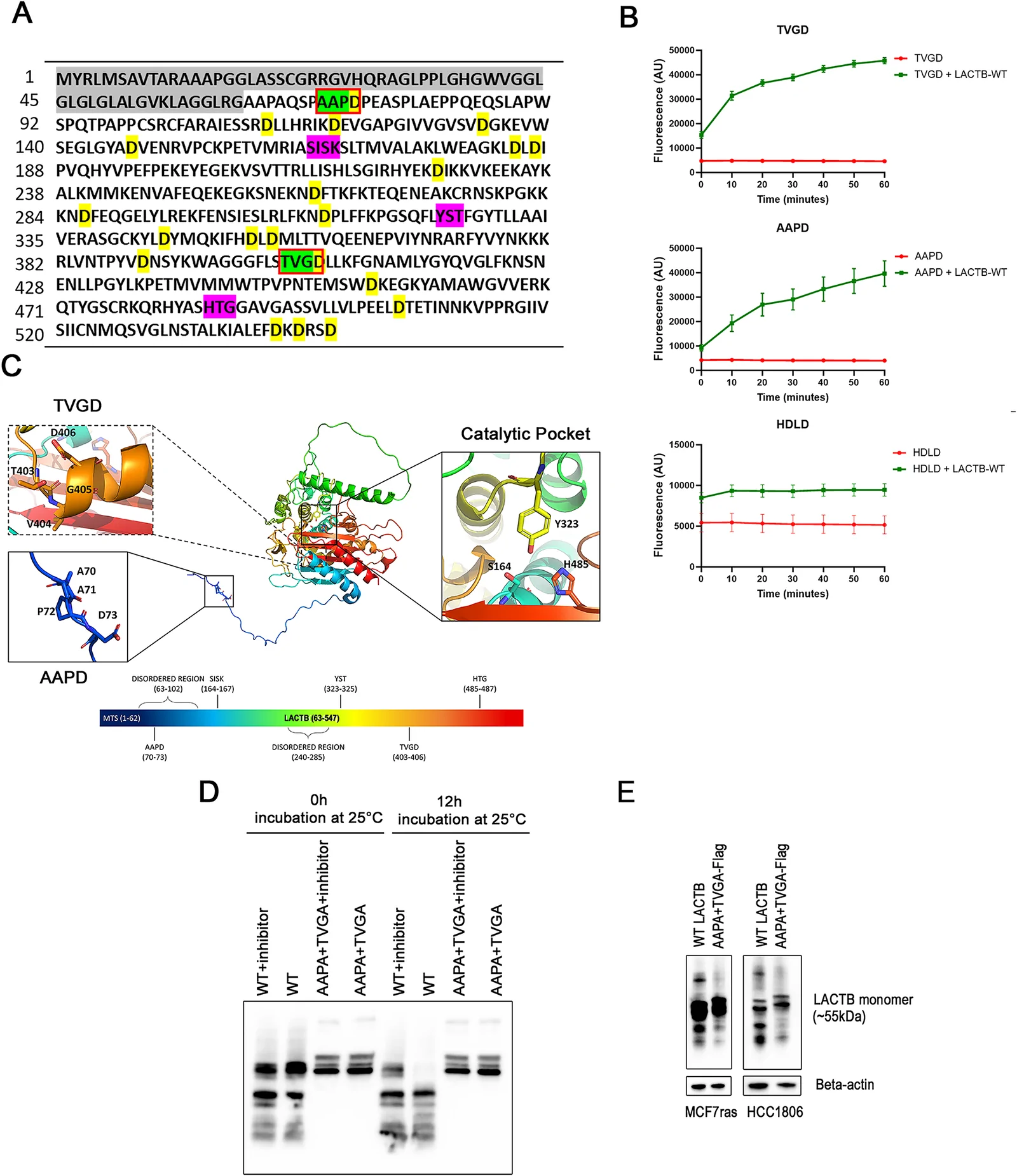
The effect of AAPD/TVGD residues on the autoproteolytic activity of LACTB. (**A**) Amino acid sequence of LACTB-WT. Gray – Mitochondria targeting sequence; Green and red – Sites cleaved by LACTB; Dark pink – Residues that constitute LACTB’s catalytic pocket; Yellow – aspartic acids within LACTB. (**B**) Enzymatic activity of LACTB-WT against the two top peptide hits–AAPD and TVGD, and against a peptide (HDLD) that did not score as a hit. The experiment was performed three times. (**C**) Schematic representation of full-length LACTB monomer structure, highlighting the locations of the catalytic pocket, as well as the targeted cleavage sites AAPD and TVGD. (**D**) Western blot analysis of recombinant LACTB-WT and recombinant LACTB-AAPA + TVGA incubated at 25 °C, in H_2_O, with or without the inhibitor Z-AAD-CMK. (**E**) Western blot analysis of LACTB-WT and LACTB-AAPA + TVGA-Flag (both induced by doxycycline for 24 h) in MCF7R and HCC1806 cells. Membrane was probed with LACTB and beta-actin antibodies. Source data are available online for this figure.

**Figure EV2:**
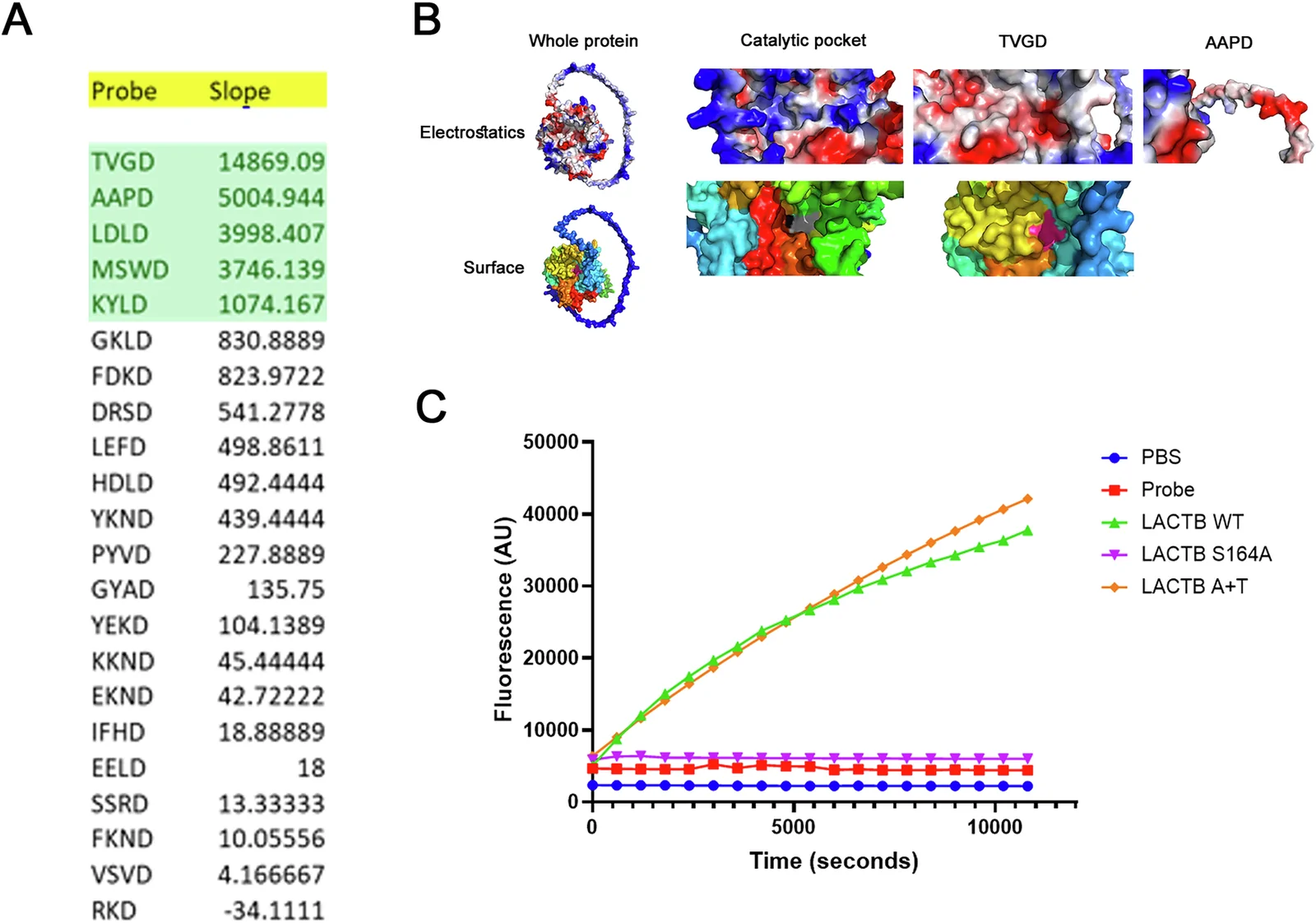
The role and position of AAPD/TVGD residues in LACTB. (**A**) The list of peptides, fused to AMC fluorescent probe, representing all tetrapeptides ending in aspartic acid within the LACTB sequence that were tested for their ability to be cleaved by LACTB. Highlighted in the table are the peptides most efficiently cleaved by LACTB, as calculated by subtracting the first fluorescence measurement from the last fluorescence measurement (slope). (**B**) Upper panel: electrostatic comparison between the catalytic pocket and TVGD and AAPD sites; Bottom panel: surface analysis of full-length LACTB-WT, showing the catalytic pocket (gray) and TVGD (magenta) regions. (**C**) Enzymatic activity of the autoproteolytic mutant LACTB-AAPA + TVGA (A + T) compared to the catalytically inactive mutant S164A and LACTB-WT. The experiment was performed twice (*N* = 2).

We wanted to examine the autoproteolytic activity of LACTB in more detail and map the amino acid residues targeted by this process. Since LACTB was shown to cleave peptides after aspartic acid residues (Keckesova et al, 2017; Bachovchin et al, 2014), we synthesized all D-end tetrapeptides from the LACTB sequence (Figs. 2A and EV2A) fused with fluorescent AMC-tag and incubated these peptides in the presence of LACTB-WT protein. AAPD and TVGD peptides were most efficiently cleaved by LACTB-WT, suggesting that these two sites might be preferentially targeted in the LACTB autoproteolytic process (Fig. 2B). AAPD is located in a structurally disordered N-terminal LACTB region, and both, TVGD and the catalytic pocket (constituted by SISK, YST, and HTG motives), are located on the surface of the LACTB molecule, which would make AAPD and TVGD sites accessible for cleavage (Fig. 2C). Regarding their charge, both AAPD and TVGD are negatively charged, as opposed to the catalytic core which is positively charged (Fig. EV2B) further supporting the notion of their possible interaction. To confirm the role of the AAPD and TVGD sites as peptide targets in the autoproteolytic activity of LACTB, we prepared a mutant of LACTB in which the aspartic acids at these two sites were mutated to alanines (LACTB-AAPA + TVGA). Our experiments showed that, as opposed to LACTB-WT, when the recombinant LACTB-AAPA + TVGA protein was incubated in vitro, it lacked the ability of autoproteolytic cleavage (as shown by the lack of degradation products), thus confirming the important role of these sites in the autoproteolytic process (Fig. 2D). Although the autoproteolytic activity is impaired in LACTB-AAPA + TVGA, this non-cleavable mutant still retains its enzymatic activity directed against a peptide probe (Fig. EV2C), suggesting that the enzymatic activity of LACTB can be directed against external peptide or against LACTB itself independently. We next investigated the behavior of this mutant in cellular assays and expressed doxycycline-inducible LACTB-WT and LACTB-AAPA + TVGA for 1 day in MCF7R and HCC1806 cells, and examined by Western blot the appearance of LACTB degradation products. Indeed, expression of LACTB-WT led to the appearance of robust degradation products, while this was not observed in the case of the non-cleavable LACTB-AAPA + TVGA mutant (Fig. 2E). The faint degradation bands present in the LACTB-AAPA + TVGA mutant samples are the result of the endogenous LACTB-WT that is present in small amounts in both MCF7R and HCC1806 cells.

### The autoproteolytic activity of LACTB influences its tumor-suppressive ability in vitro and in vivo

**Figure EV3:**
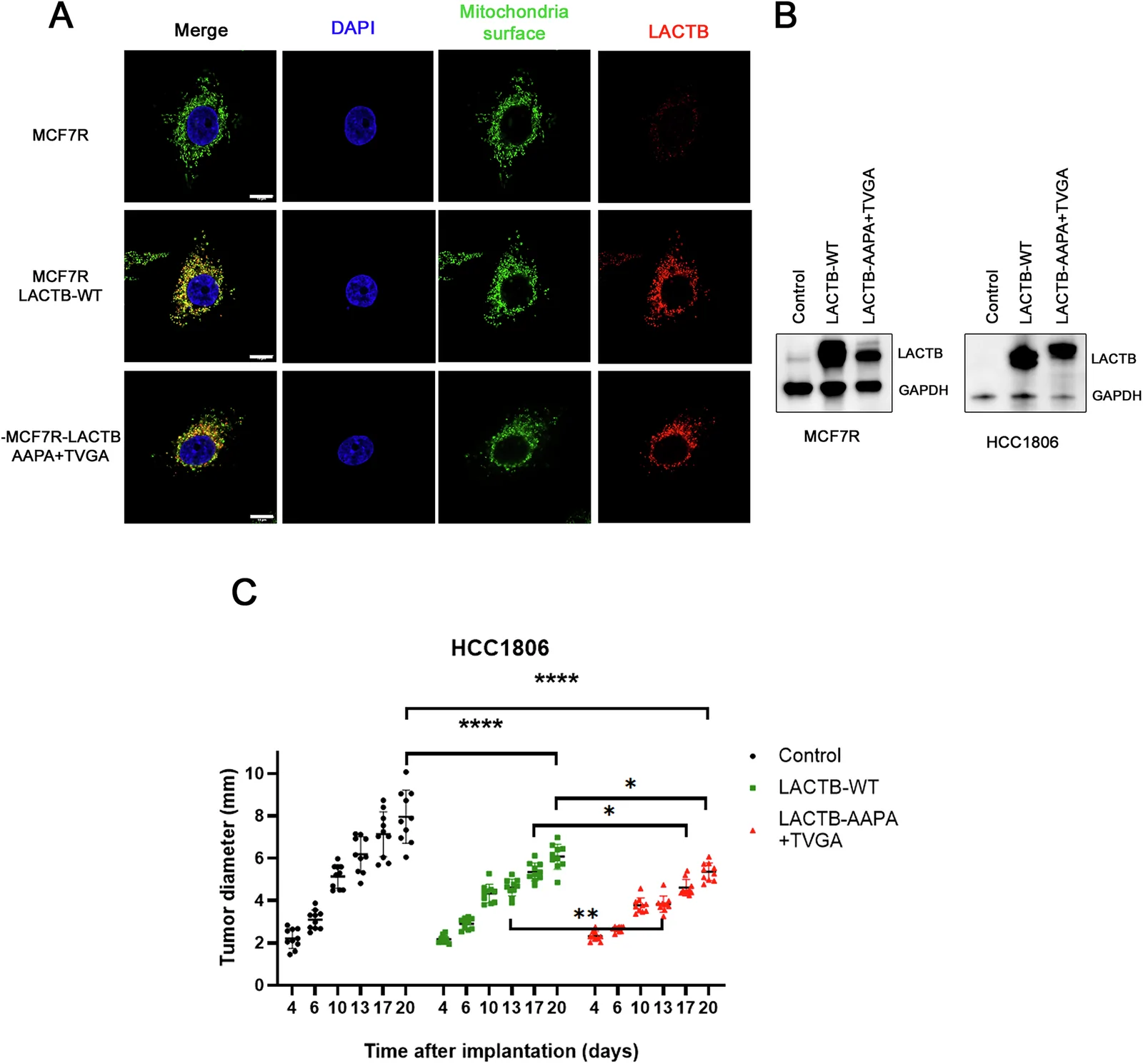
Expression and effect of LACTB-AAPA+TVGA in cells and tumors. (**A**) Confocal microscopy images of the localization of LACTB-WT and LACTB-AAPA + TVGA in MCF7R, after one day of induction. Blue corresponds to DAPI, green labels mitochondria surface (Merck, MAB1273), and red represents LACTB. Scale bar is 5 µm. (**B**) Expression levels of LACTB-WT and LACTB-AAPA + TVGA in MCF7R and HCC1806 cells. (**C**) Growth of tumors formed by injection of control HCC1806 (black), HCC1806 LACTB-WT (green), and HCC1806 LACTB-AAPA + TVGA (red) cells into the mouse mammary fat pad. Doxycycline treatment was initiated at 6 days of tumor growth (blue arrows). Statistics were calculated using two-way ANOVA, *p < 0.05; **p < 0.01, ****p < 0.0001. *N* = 10. Mean ± SD. *p < 0.05; **p < 0.01, ****p < 0.0001. HCC1806 control vs HCC1806_LACTB at 20 days, *p* = 1.72 × 10^−11^; HCC1806 control vs HCC1806_LACTB-AAPA + TVGA at 20 days, *p* = 7.3 × 10^−19^; HCC1806_LACTB vs HCC1806_LACTB-AAPA + TVGA at 20 days, *p* = 0.0147; HCC1806_LACTB vs HCC1806_LACTB-AAPA + TVGA at 17 days, *p* = 0.0105; HCC1806_LACTB vs HCC1806_LACTB-AAPA + TVGA at 13 days, *p* = 0.0065.

**Figure 3:**
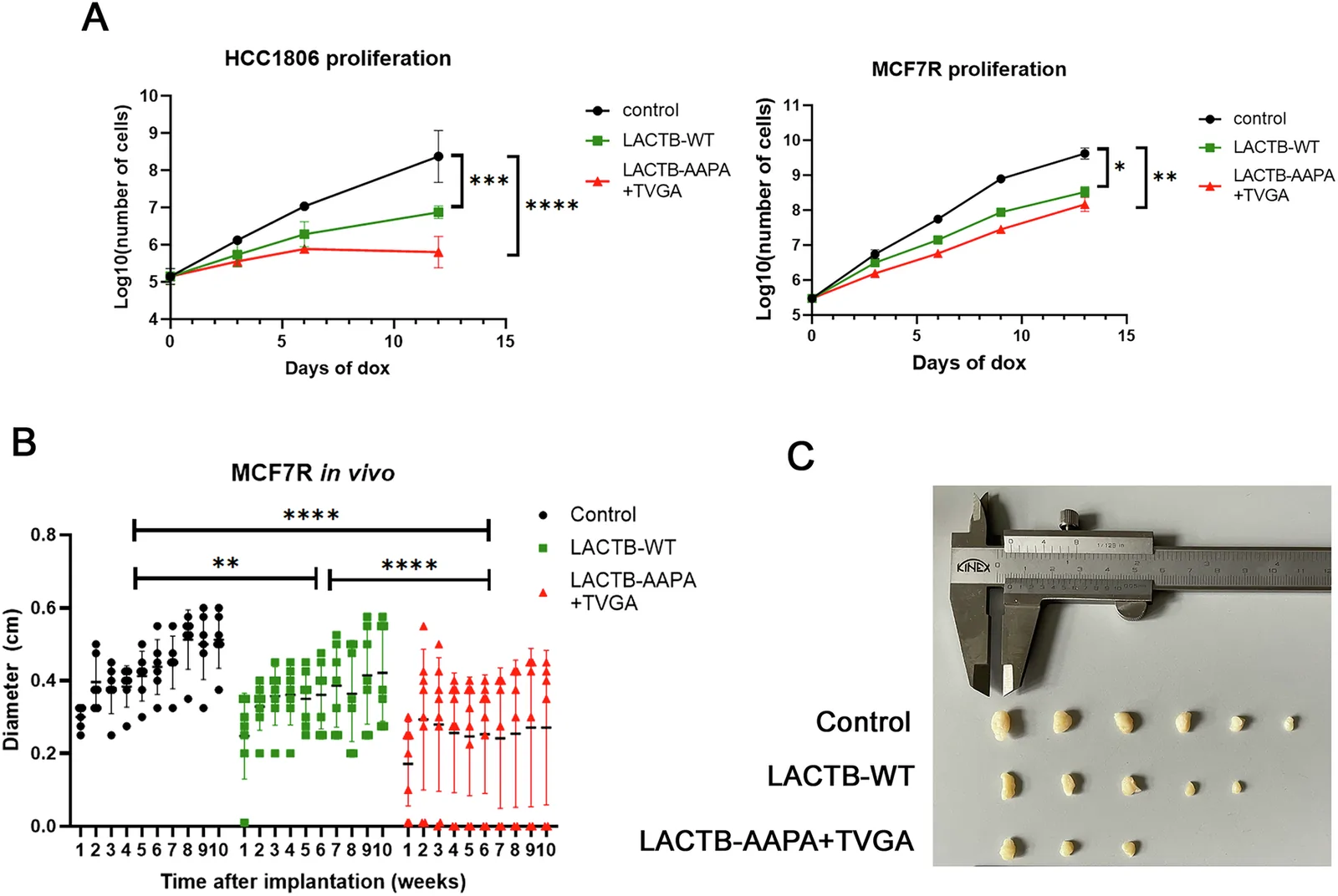
The autoproteolytic activity of LACTB influences its tumor-suppressive ability in vitro and in vivo. (**A**) Proliferation curve of MCF7R and HCC1806 cells upon expression of dox-inducible LACTB-WT and LACTB-AAPA + TVGA. Cell numbers were determined by counting live cells every 3 days up to day 12. ns *p* > 0.05; *p < 0.05; **p < 0.01; ***p < 0.001, ****p < 0.0001. HCC1806 control vs HCC1806 LACTB-WT, *p* = 0.0009; HCC1806 control vs HCC1806 LACTB-AAPA + TVGA, *p* = 5.4 × 10^−6^; MCF7R control vs MCF7R-LACTB-WT, *p* = 0.0483; MCFR control vs MCF7R-LACTB-AAPA + TVGA, *p* = 0.0043. (**B**) Growth of tumors formed by injection of MCF7R (black), MCF7R-LACTB-WT (green), and MCF7R-LACTB-AAPA + TVGA (red) cells into the mouse mammary fat pad. Doxycycline treatment was initiated at 2 weeks of tumor growth (blue arrows). Statistics were calculated comparing the mean of each group using a two-way ANOVA test; **p < 0.01; ****p < 0.0001. MCF7R vs. MCF7R-LACTB-WT, *p* = 0.0083. MCF7R vs. MCF7R-LACTB-AAPA + TVGA, *p* = 4.76×10^−12^. MCF7R-LACTB-WT vs. MCF7R-LACTB-AAPA + TVGA, *p* = 1.10 × 10^−5^. (**C**) Comparison of tumor sizes between MCF7R, MCF7R-LACTB-WT, and MCF7R-LACTB-AAPA + TVGA. Some tumors in the MCF7R-LACTB-AAPA + TVGA group were completely eliminated during doxycycline treatment. Source data are available online for this figure.

We examined whether the autoproteolytic activity of LACTB has physiological significance and whether it can modulate LACTB’s tumor-suppressive activity. We therefore expressed the non-cleavable LACTB-AAPA + TVGA and LACTB-WT in MCF7R and HCC1806 cells. Using Western blot analysis and confocal microscopy, we confirmed that the LACTB-AAPA + TVGA mutant is stably expressed in cells, is correctly localized in mitochondria, and its levels of expression are comparable to those of LACTB-WT in HCC1806 cells and are slightly decreased in MCF7R cells (Fig. EV3A,B). We then monitored the growth of MCF7R and HCC1806 cells upon induction of these proteins for up to 12 days. Interestingly, the non-cleavable LACTB-AAPA + TVGA mutant was even more potent at inhibiting cancer cell growth than its wild-type counterpart in both cell types (Fig. 3A). We then investigated whether LACTB-AAPA + TVGA expression negatively affects tumor growth. Control MCF7R cells and MCF7R cells transduced with dox-inducible LACTB-WT or with dox-inducible LACTB-AAPA + TVGA were injected orthotopically into mice, and the growth of the tumors was monitored after the induction of both proteins. Tumors in which non-cleavable LACTB-AAPA + TVGA had been induced decreased in size even more significantly than tumors with LACTB-WT (Fig. 3B,C), confirming the role of autoproteolytic activity in LACTB’s tumor-suppressive effects. Similar results were observed with HCC1806 tumors (Fig. EV3C). These results suggest that LACTB protein impaired in its autoproteolytic ability is even more potent in negatively affecting cancer cells in vitro and in vivo than LACTB-WT.

### MRPS34 is an interacting partner of LACTB

**Figure 4:**
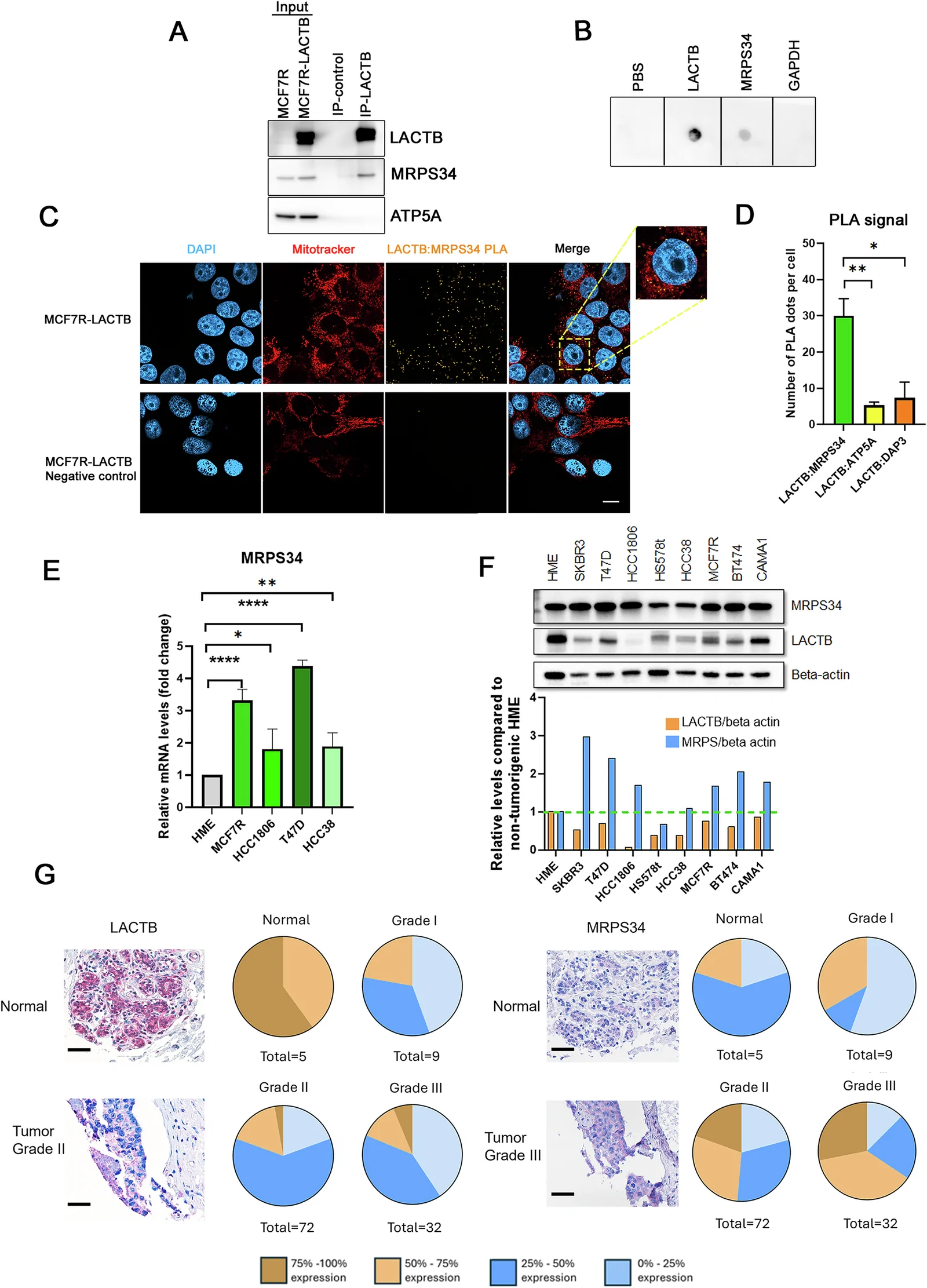
LACTB is in complex with MRPS34. (**A**) Immunoprecipitation (IP) experiments through LACTB antibody followed by Western blot analysis with antibodies against LACTB, MRPS34, and ATP5A. The input consists of lysate of MCF7R and MCF7R overexpressing doxycycline-inducible LACTB for 1 day. IP-control was done by immunoprecipitating with Resistin IgG antibody. Part of this figure is shared with Fig. EV4A. (**B**) Dot blot assay with LACTB, MRPS34, and GAPDH recombinant proteins, followed by incubation with recombinant LACTB-WT and immunoblotting with LACTB antibody. (**C**) PLA assay performed in MCF7R-LACTB cells, using LACTB and MRPS34 primary antibodies. DAPI is depicted in blue, mitochondria are represented in red (stained with MitoTracker Deep Red), and yellow indicates the positive PLA signal (dots represent in situ interactions). Scale bar is 10 µm. (**D**) Quantification of the number of PLA dots per cell. The experiment was performed in MCF7R cells and used the following antibody combinations: LACTB-MRPS34, LACTB-ATP5A, and LACTB-DAP3. Statistics were performed using an unpaired Student’s *t*-test from three different fields. *N* = 3. Mean ± SEM is represented, *p < 0.05; **p < 0.01. (**E**) mRNA expression of endogenous MRPS34, normalized to GAPDH, in non-tumorigenic HME, and in cancer cell lines MCF7R, HCC1806, HCC38 and T47D. Mean ± SEM is represented. *N* = 5. Statistics were calculated using an unpaired *t*-test, *p < 0.05; **p < 0.01; ****p < 0.0001. (**F**) Protein expression of endogenous MRPS34 and LACTB in a panel of breast cancer cells and non-tumorigenic HME cells. Graphs show the quantification of MRPS34 and LACTB intensities, normalized to beta-actin intensity, for each cell line, relative to non-tumorigenic HME cells. A representative experiment is shown. All experiments were performed three times (*N* = 3). (**G**) Immunohistology staining of human breast clinical tissues (*N* = 119) of normal or tumorigenic (grade 1, 2, 3) origin for LACTB and MRPS34 protein expression. Scale bar =  50 µm. Source data are available online for this figure.

**Figure EV4:**
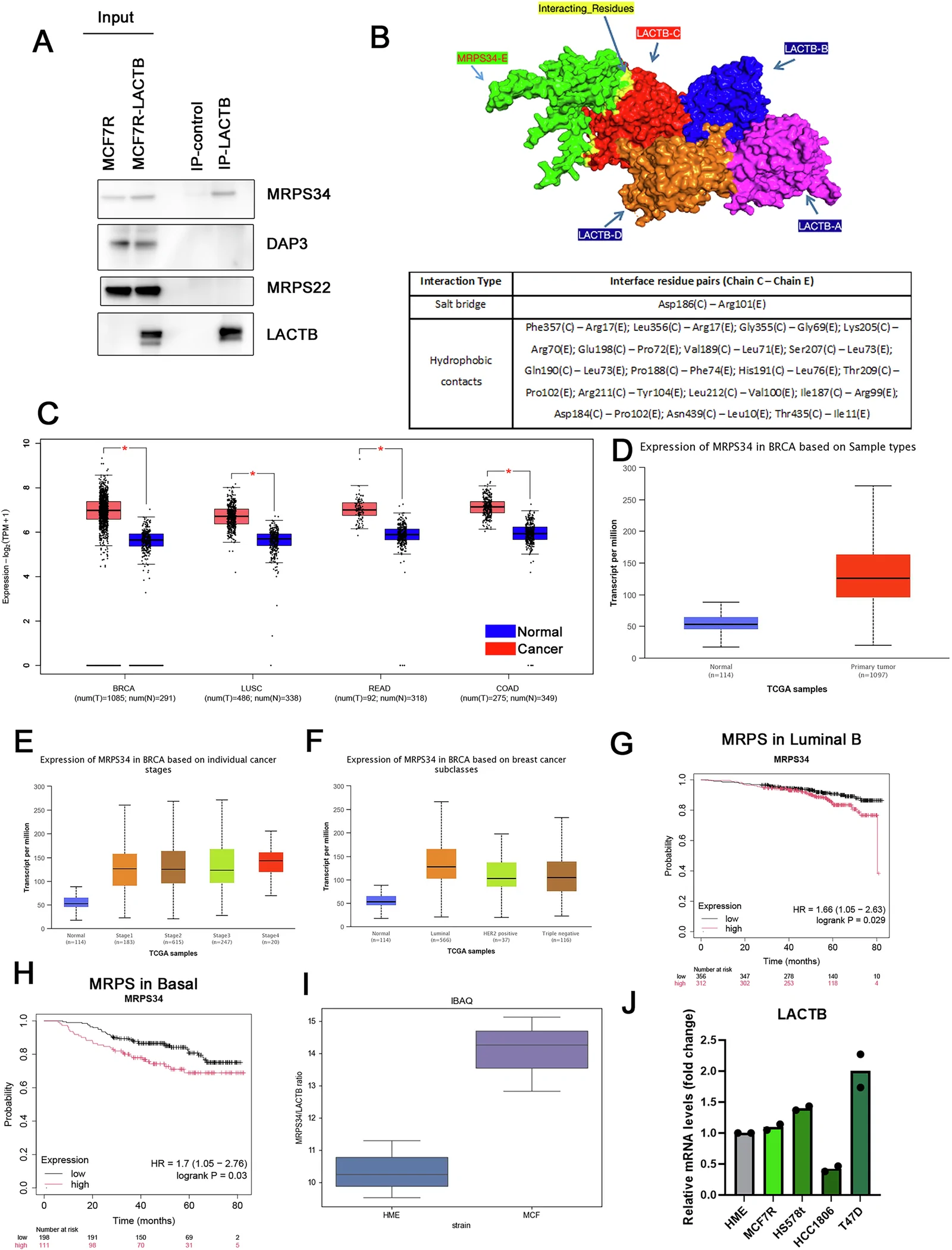
Association between MRPS34 and LACTB. (**A**) Immunoprecipitation (IP) through LACTB antibody followed by Western blot with antibodies against MRPS34, DAP3/MRPS29, and MRPS22. The input consists of lysate of MCF7R and MCF7R overexpressing doxycycline-inducible LACTB for 1 day. IP-control was done by immunoprecipitating with Resistin IgG antibody. Part of this figure is shared with Fig. 4A. (**B**) Structural depiction of the interaction between the LACTB tetramer and MRPS34. The LACTB subunits (chains A–D) are illustrated in magenta, blue, orange, and red, respectively, while MRPS34 (chain E) is shown in green. Residues involved in interfacial contacts are highlighted in yellow. The accompanying table details the interface interactions, categorizing residue pairs into salt bridges and hydrophobic contacts. (**C**) Expression of MRPS34 in normal (blue) and cancer (red) tissues of breast (cancer *N* = 1085; normal *N* = 291, *p* = 1.62 × 10^−12^), lung (cancer *N* = 486; normal *N* = 338, *p* = 1.62 × 10^−12^), rectum (cancer *N* = 92; normal *N* = 318, *p* = 1.64 × 10^−12^), and colon cancer (cancer *N* = 275; normal *N* = 349, *p* = 1.62 × 10^−12^). The graph was obtained using the GEPIA2 tool. Box plots show the median (center line), 25th–75th percentiles (box bounds), and whiskers indicate the minimum and maximum values. (**D**–**F**) Expression of MRPS34 in normal and breast cancer (BRCA) tissues stratified by different stages and subtypes. Graph obtained using the UALCAN tool. In panel (**D**), normal *N* = 114, primary tumor *N* = 1097; in panel (**E**), normal *N* = 114, stage 1 *N* = 183, stage 2 *N* = 615, stage 3 *N* = 247 and stage 4 *N* = 20; in panel (**F**), normal *N* = 114. Luminal *N* = 566, HER2 positive *N* = 37 and triple negative *N* = 116. (**G**) Effects of the expression of MRPS34 on overall survival in patients with luminal B breast cancer, using the KMplotter tool. (**H**) Effects of MRPS34 expression on overall survival in patients with basal breast cancer, using the KMplotter tool. (**I**) Mass spectrometry IBAQ analysis examining the stoichiometry of MRPS34 and LACTB in non-tumorigenic HME cells and tumorigenic MCF7ras cells. Data of three replicates is shown (*N* = 3). (**J**) RT-PCR analysis of endogenous LACTB levels, normalized to GAPDH, in non-tumorigenic HME cells, and in cancer cell lines MCF7R, HCC1806, T47D and HCC38. Samples were shared with Fig. 4E, where we assessed the MRPS34 levels. Mean ± SD. The experiment was performed twice (*N* = 2).

LACTB has been shown by many studies to be efficiently downregulated in cancer cells (Keckesova et al, 2017; Zeng et al, 2018; Wang et al, 2020; Li et al, 2019; Cutano et al, 2022), and our results above suggest that LACTB may be downregulated by cancer cells through induction of its autoproteolytic activity. This prompted us to research this possible regulatory mechanism and to identify the regulator (s) of LACTB’s autoproteolytic activity in cancer cells using immunoprecipitation assays. In these experiments, we pulled down LACTB protein complexes from MCF7R cells expressing endogenous LACTB or doxycycline-inducible wild-type LACTB (1-day induction) and analyzed them by LC-MS/MS for the presence of LACTB’s binding partners. The proteins identified by mass spectrometry (Dataset EV2) were then validated for their ability to bind LACTB via secondary immunoprecipitation and subsequent Western Blot analysis. The results showed that mitochondrial small ribosomal subunit protein mS34 (MRPS34) was efficiently pulled down through LACTB antibody (Fig. 4A). Within mitochondria, MRPS34 helps to stabilize the structure of the small ribosomal subunit and is essential for the efficient translation of mitochondrial-encoded genes. We did not observe any interaction of LACTB with other tested members of the MRPS-family, such as DAP3 (MRPS29) or MRPS22 (Fig. EV4A). The interaction of LACTB and MRPS34 proteins was further confirmed in vitro by subsequent dot blot experiments (Fig. 4B) and in a cellular context by proximity ligation assay (PLA) in MCF7R cells (Fig. 4C,D). PLA was also performed using antibodies against other mitochondrial proteins (such as ATP5A and DAP3), which, unlike MRPS34, showed no interaction with LACTB (Fig. 4D). Using LZerD software, we performed protein docking to predict the interaction site between LACTB and MRPS34. The predicted models, which will require further experimental validation in our follow-up work, show binding of MRPS34 to LACTB tetramer and the formation of a potential salt bridge between Asp186 (LACTB) and Arg101 (MRPS34) (Fig. EV4B). We next investigated the expression pattern of MRPS34 in normal and cancer cells. Bioinformatic analysis showed that tumor types (such as breast, lung, colon), where LACTB was previously reported to be downregulated (Keckesova et al, 2017; Zeng et al, 2018; Wang et al, 2020; Xu et al, 2022), have a significantly higher expression of MRPS34 compared to normal tissues (Fig. EV4C); in breast cancer, this upregulation occurs throughout all stages, subclasses, and types (Fig. EV4D–F). Moreover, patients with higher MRPS34 expression tend to have a lower survival rate (Fig. EV4G,H). RT-qPCR analysis confirmed that MRPS34 is upregulated at mRNA levels also in breast tumorigenic cell lines such MCF7R, HCC1806, T47D and HCC38 when compared to non-tumorigenic HME cells (Fig. 4E). We further profiled the protein expression pattern of endogenous MRPS34 and LACTB by Western blot in a panel of eight breast cancer cell lines and found that MRPS34 protein is upregulated and LACTB is downregulated in majority of the tested cancer cell lines compared to their levels in HME cells (Fig. 4F). This was confirmed by our mass spectrometry stoichiometry analysis where ratio of MRPS34 to LACTB proteins was shown to be higher in tumorigenic MCF7ras cells when compared to non-tumorigenic HME cells (Fig. EV4I; Dataset EV3). The RT-qPCR analysis of LACTB mRNA levels in several tumorigenic cell lines showed no strong correlation with its downregulation on a protein level, suggesting that LACTB is, in these cell lines, downregulated on a post-transcriptional level (Fig. EV4J). Furthermore, we extended our experiments to human breast clinical tissues (*n* = 119) of normal or tumorigenic (grade 1, 2, 3) origin. Immunohistology staining showed that tissues where LACTB protein was downregulated had increased expression of MRPS34 (Fig. 4G). Thus, all these sets of experiments showed an interaction between LACTB and MRPS34 and pointed to an inverse expression pattern of LACTB and MRPS34 in human cell lines and tissues.

### MRPS34 is a negative regulator of LACTB through enhancement of its autoproteolytic activity

**Figure 5:**
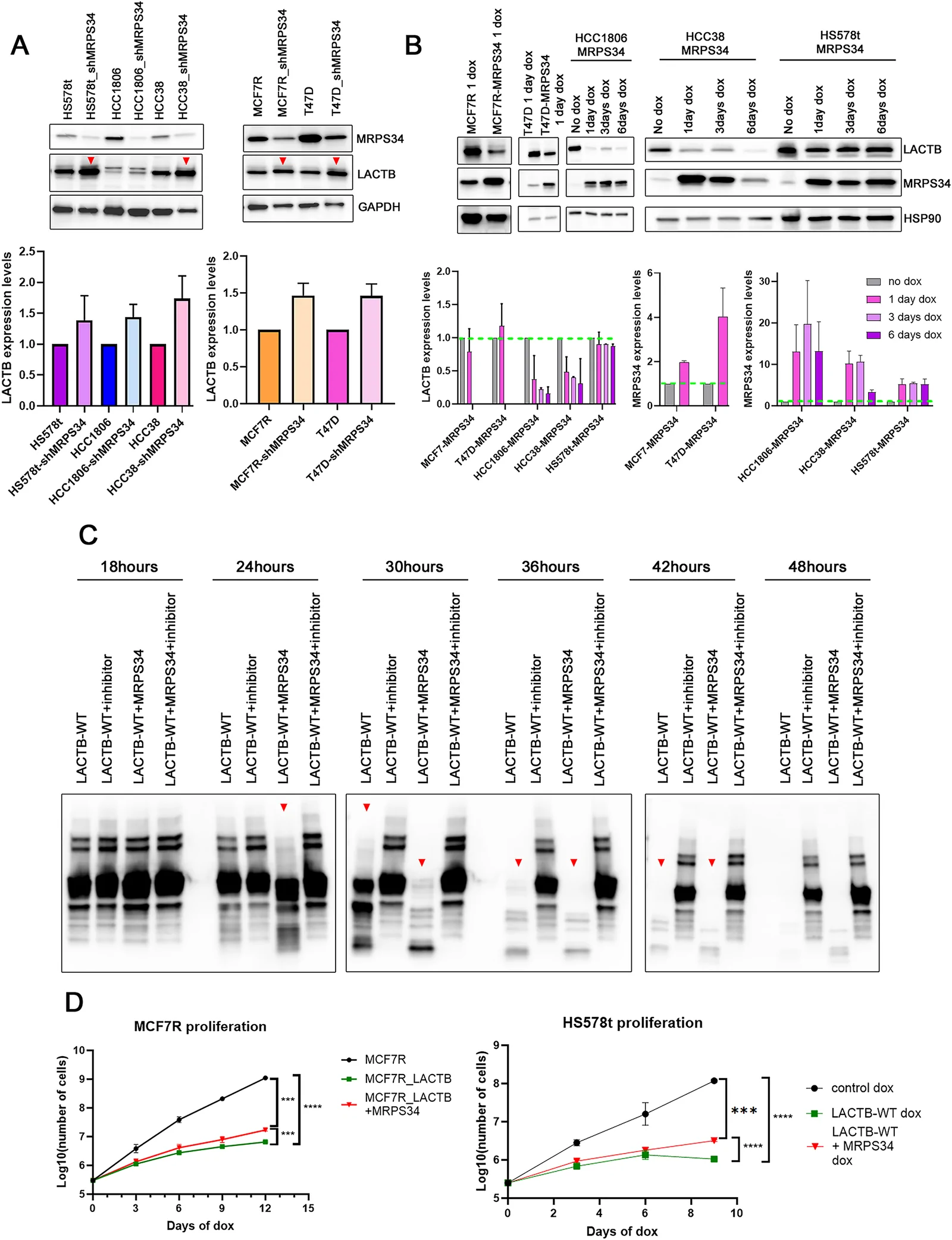
MRPS34 is a negative regulator of LACTB. (**A**) LACTB protein expression levels in control cells and upon downregulation of MRPS34 in a panel of tumorigenic (MCF7R, T47D, HS578T, HCC1806, and HCC38) cell lines. Graphs show the quantification of monomer LACTB intensity, normalized to GAPDH intensity for each cell line, followed by normalization of shMRPS34 to the respective control cells. The experiment was performed three times (*N* = 3). Mean ± SD. Arrowheads show the increase in the LACTB levels. (**B**) LACTB expression levels in control cells and upon overexpression of dox-inducible MRPS34, in a panel of tumorigenic (MCF7R, T47D, HCC1806, and HS578T, HCC38) cell lines. Graphs represent the quantification of MRPS34 or of the monomer LACTB intensity normalized to the GAPDH (in MCF7R) or HSP90 (in T47D, HCC1806, HCC38, and HS578T) intensity for each cell line. The green perforated line shows the level of MRPS34 or LACTB in control cells. The experiment was performed three times (*N* = 3). Mean ± SD is represented. (**C**) LACTB and LACTB + MRPS34 recombinant proteins, with and without LACTB inhibitor, were incubated at 25 °C for 18–48 h in H_2_O, followed by Western blot and immunoblotting with LACTB antibody. Arrowheads showing the decrease of the LACTB levels in multimer and monomer form. (**D**) Proliferation rate of MCF7R and HS578T cells with and without dox-inducible LACTB and MRPS34, upon doxycycline induction for up to 12 days. The number of cells is shown on a logarithmic scale. The experiment was performed three times for MCF7R cells and 2 times for HS578T cells. Mean ± SD. ***p < 0.001; ****p < 0.0001. MCF7R vs MCF7R_LACTB, *p* = 6.99 × 10^−15^; MCF7R vs MCF7R_LACTB + MRPS34, *p* = 1.25 × 10^−13^; MCF7R_LACTB vs MCF7R_LACTB + MRPS34, *p* = 0.0008. HS5787 vs HS578T_LACTB, *p* = 2.30 × 10^−10^; HS578T vs HS578T_LACTB + MRPS34, *p* = 5.13 × 10^−9^; HS578T_LACTB vs HS578T_LACTB + MRPS34, *p* = 0.0010. Source data are available online for this figure.

**Figure EV5:**
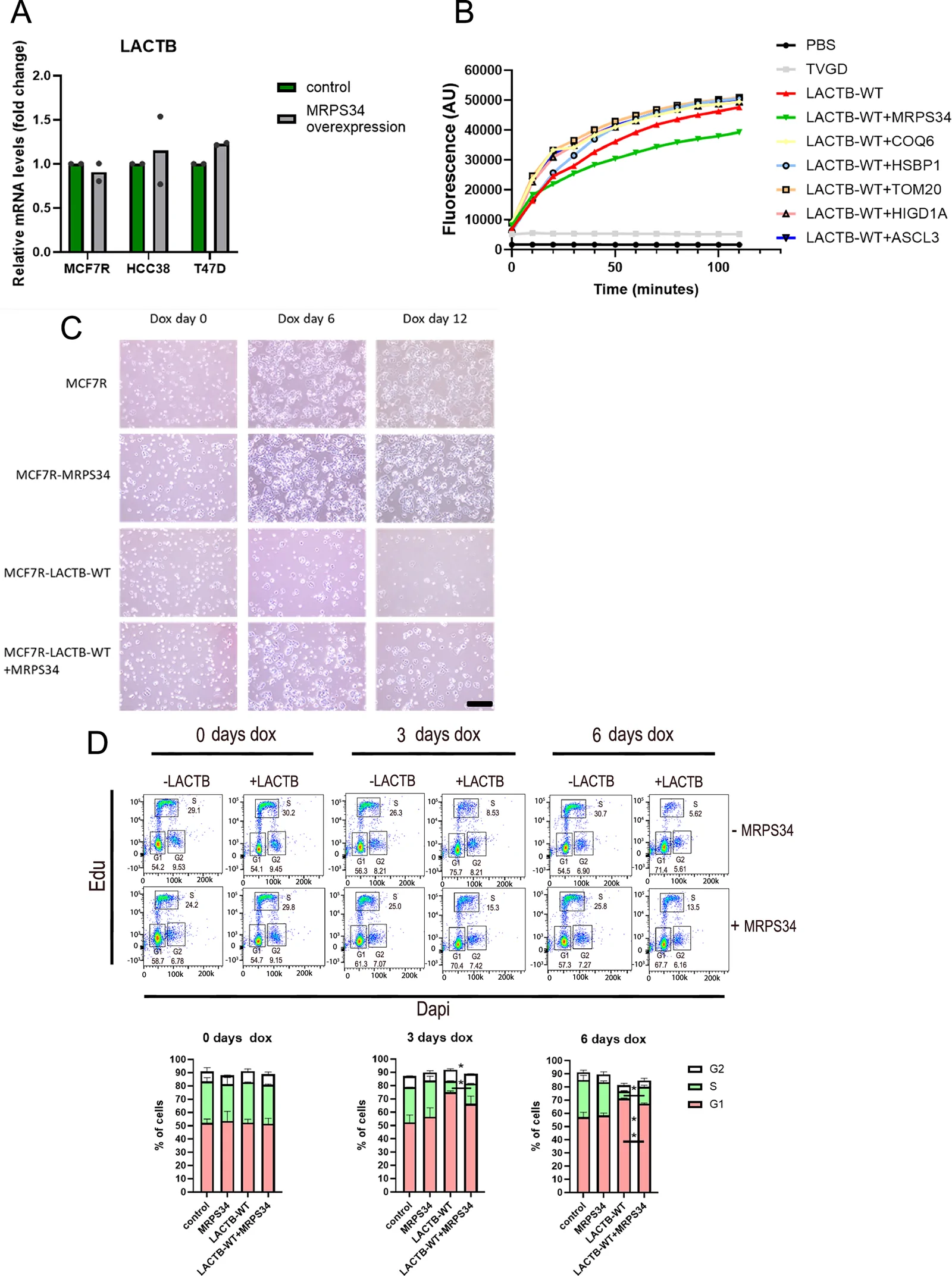
MRPS34 negatively affects LACTB in cells. (**A**) Endogenous LACTB mRNA levels in MCF7R, HCC38, and T47D cancer cells with or without MRPS34 induction for 1 day, normalized to GAPDH. The experiment was performed twice (*N* = 2). Mean ± SD (**B**) Enzymatic activity of LACTB directed against a peptide probe in the presence of MRPS34 and other mitochondrial proteins. LACTB-WT was pre-incubated with the respective proteins for 48 h at 25 °C prior to the assessment of its enzymatic activity. (**C**) Light microscopy images of MCF7R cells, expressing LACTB, MRPS34, or both for 0, 6, and 12 days. Scale bar: 500 µm. (**D**) Flow cytometry-based cell cycle analysis of EdU/DAPI in MCF7R cells. Comparison of the DNA synthesis (S phase) and G1 phase in MCF7R-LACTB-WT and MCF7R-LACTB-WT + MRPS34 cells upon doxycycline induction (day 0, 3, and 6), using Student’s *t*-test. Experiments were performed three times (*N* = 3). Significance was reached when *P* < 0.05. *p < 0.05; **p < 0.01. Mean ± SD represented. *p < 0.05; **p < 0.01. MCF7R-LACTB-WT vs MCF7R MRPS34 3 days dox, *p* = 0.0022; S phase of MCF7R-LACTB-WT vs MCF7R MRPS34 6 days dox, *p* = 0.0187; G1 phase of MCF7R-LACTB-WT vs MCF7R MRPS34 6 days dox, *p* = 0.0040.

To further investigate the role of MRPS34 in LACTB regulation, we employed MRPS34 shRNA in a panel of tumorigenic breast cancer cell lines (MCF7R, T47D, HS578T, HCC1806, and HCC38) and monitored endogenous LACTB levels upon MRPS34 downregulation. We observed that downregulation of MRPS34 led to increases in endogenous LACTB protein levels in the majority of the tumorigenic cell lines studied, revealing MRPS34’s role in negatively regulating LACTB levels in breast cancer cells (Fig. 5A). Of note, strong MRPS34 knockdown had a toxic effect on several cancer cell lines; therefore, we aimed for partial knockdown of MRPS34. Even under these conditions, significant increases in LACTB expression levels were readily observed. We next overexpressed MRPS34 protein in MCF7R, T47D, HCC1806, HS578T, and HCC38, and noticed robust decreases of endogenous LACTB levels in cell lines tested (Fig. 5B). We did not observe any difference in LACTB mRNA levels in MCF7R, HCC38 and T47D cells overexpressing MRPS34 when compared to the control cells, suggesting that the negative regulation of LACTB protein levels by MRPS34 occurs at a post-transcriptional level (Fig. EV5A). Since our previous results showed that LACTB can degrade itself through autoproteolytic activity and that MRPS34 is a negative regulator of LACTB, we investigated the possibility whether the MRPS34 exerts its negative regulation of LACTB by enhancing its autoproteolytic activity. We therefore incubated recombinant LACTB-WT with and without MRPS34 for different time points, from 18 to 48 h. MRPS34’s presence significantly accelerated the autoproteolytic activity of LACTB, which was observable at early time points of co-incubation (Fig. 5C). Of note, we noticed that the disappearance of LACTB in the presence of MRPS34 was more apparent and appeared at earlier time points in the multimeric LACTB forms. In the presence of the inhibitor of the enzymatic activity of LACTB, no autoproteolytic activity was observed at any of the time points (Fig. 5C). Regarding the enzymatic activity efficiency directed against the peptide probe, recombinant LACTB incubated together with MRPS34 displayed a decrease in the enzymatic activity (Fig. EV5B), confirming the onset of the accelerated degradation of LACTB-WT in the presence of MRPS34. However, when recombinant LACTB was incubated with other mitochondrial proteins (COQ6, HSBP1, TOM20, HIGD1A, and ASCL3), there was no decrease in its enzymatic activity (Fig. EV5B), confirming the specific effect MRPS34 has on LACTB biology. We next assessed whether MRPS34 overexpression could affect the proliferation rate of breast cancer cells with concomitant LACTB expression. The results showed that LACTB-induced decrease in cell proliferation in MCF7R and HS578 T cells was indeed partially recovered by concomitant MRPS34 induction (Figs. 5D and EV5C), confirming the negative regulation of LACTB by MRPS34 and the resulting positive effect on cancer cell viability upon MRPS34 induction. Similarly, flow cytometry analysis examining the cell cycle progression in MCF7R cells showed that overexpression of MRPS34 in LACTB-induced cells partly restores G1 cell cycle progression and significantly increased S phase content when compared to LACTB-induced cells without MRPS34 (Fig. EV5D), further confirming that expression of MRPS34 counteracts the tumor-suppressive effects of LACTB.

Overall, these results demonstrate that MRPS34 acts as a negative regulator of LACTB by enhancing its autoproteolytic activity, thereby promoting cancer cell survival.

## Discussion

**Figure 6:**
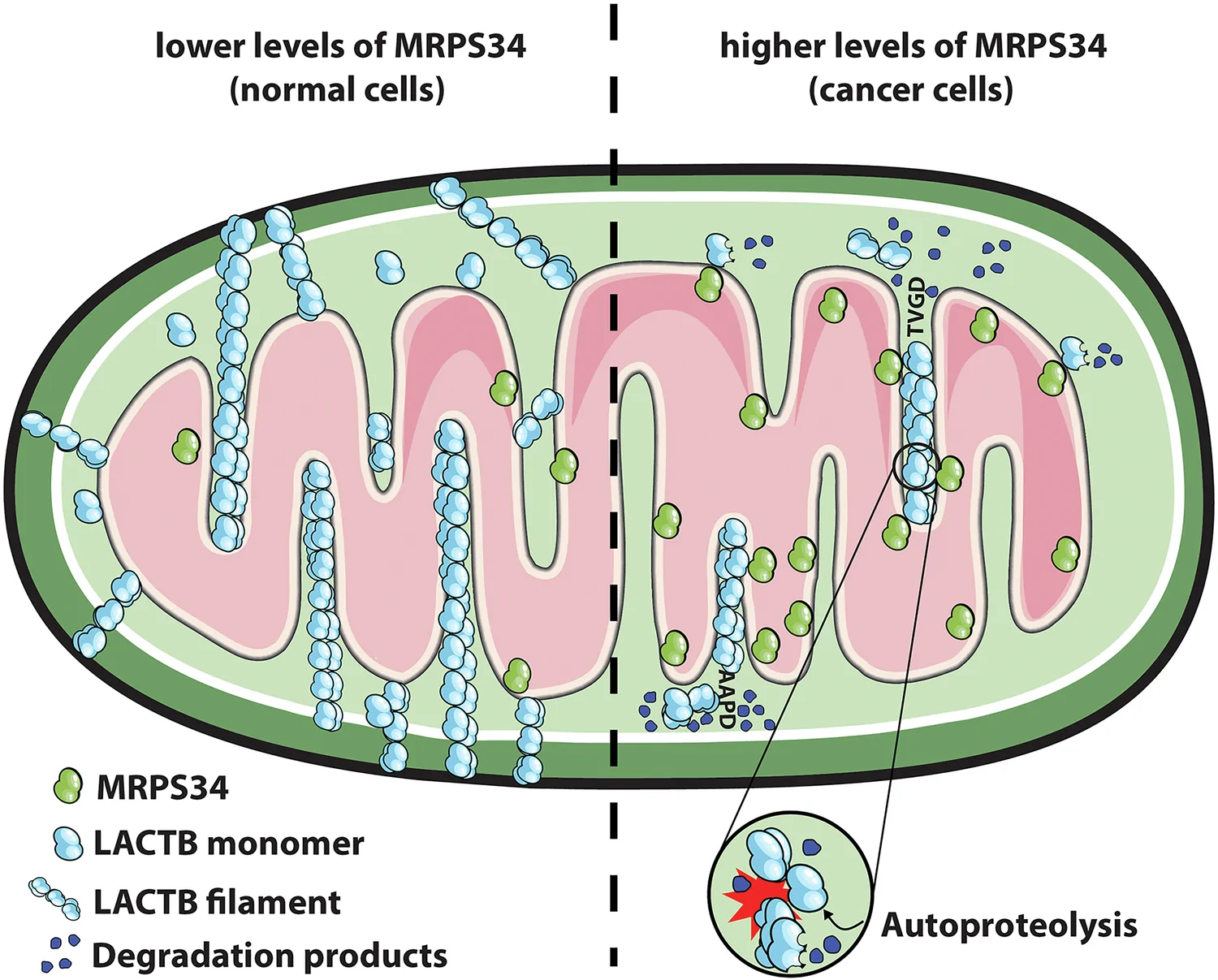
Graphic representation of LACTB’s mechanism of regulation in breast cancer cells. In normal non-tumorigenic cells, the levels of MRPS34 are low, and LACTB is present in abundance in the form of monomers and/or multimers/filaments mainly in the intermembrane mitochondrial space. In cancer cells, the levels of MRPS34 are upregulated. MRPS34 interacts with LACTB, which leads to the onset of its autoproteolytic activity. This activity is primarily directed against AAPD and TVGD sequences within LACTB and leads to its self-degradation, resulting in lower levels of LACTB in cancer cells.

In this study, we uncovered the mechanisms cancer cells use to post-translationally downregulate the LACTB tumor suppressor, thereby keeping its expression levels low. Through our search for a LACTB substrate, we showed that LACTB possesses autoproteolytic activity that is enhanced by mitochondrial MRPS34 protein, which, through interaction with LACTB, acts as a negative regulator of LACTB. The LACTB-MRPS34 axis is disrupted by cancer cells, which, through upregulation of MRPS34, achieve efficient downregulation of LACTB via its own autodegradation (Fig. 6).

Mammalian mitochondria contain mitoribosomes, which consist of a small (28S) and large (39S) subunits, both containing mtDNA-encoded rRNA—mainly located in the interior of the mitoribosome, and nuclear-encoded mitochondrial ribosomal proteins (MRPs)—present on the mitoribosome surface (Lopez Sanchez et al, 2021; Amunts et al, 2015; Englmeier et al, 2017). The development of many cancers has been associated with enhanced expression of MRPs, which could be explained by the increased metabolic activity of cancer cells (Huang et al, 2020). LACTB has previously been associated with the large subunit of the mammalian mitochondrial ribosome (Koc et al, 2001), and our study shows that it also interacts with MRPS34, the small subunit of the mitoribosome, which is localized in the mitochondrial matrix and tightly tethered to the inner mitochondrial membrane (Greber and Ban, 2016). Later studies showed that LACTB is also localized in the mitochondrial intermembrane space, where it forms filaments that are suggested to be embedded in the inner mitochondrial membrane (Polianskyte et al, 2009). Therefore, it is plausible for LACTB and MRPS34 to interact in the mitochondrial matrix or within the inner mitochondrial membrane area.

We speculate that, under normal physiological conditions, the autoproteolytic activity of LACTB serves as a regulatory mechanism for mitochondrial LACTB protein levels or its maturation stages. LACTB, through its ability to form filaments within the intermembrane mitochondrial space, was suggested to play a role in the structural organization of mitochondria. It is possible that the autoproteolytic processing of LACTB might, through changes in the ratios of LACTB monomers and LACTB filaments, serve as a fast and efficient mitochondrial restructuring and adaptation mechanism to various cellular stimuli (modulation of differentiation, mitochondrial pore permeability, metabolism, ion accessibility, or signaling pathways stemming from mitochondria). Our previous work (Baudysova, 2019) showed the sensitivity of LACTB’s enzymatic activities to pH, buffers, salts, and ions, suggesting that LACTB might serve either as a sensor of these conditions within mitochondria or be regulated by them to fulfill distinct mitochondrial functions. Depending on the nature of these conditions, LACTB, through switching between the monomeric and/or filamentous forms or between enzymatic activity directed against the cellular substrate or itself, influences the structure of the mitochondria and the processes realized within them.

Overall, our study identified novel mechanisms of tumor suppressor regulation by cancer cells and uncovered additional vulnerabilities, which might help us in our fight against cancer progression.

## Methods

**Table:** Reagents and tools table

Reagent/resource	Reference or source	Identifier or catalog number
**Experimental models**		
Adult female NSG	The Jackson Laboratory	(NOD.Cg-Prkdcscid Il2rgtm1Wjl/SzJ)
MCF7/R	Merck	CVCL_0063
HMEC/HME	Lonza	CVCL_3383
HEK293T	ATCC	CVCL_0063
HCC38	ATCC	CVCL_1267
HS578T	ATCC	CVCL_0332
T47D	ATCC	CVCL_0553
SKBR3	ATCC	CVCL_0033
CAMA1	ATCC	CVCL_1115
BT474	ATCC	CVCL_0179
**Recombinant DNA**		
FUW-LACTB	Dr. Robert Weinberg, MIT	
FUW-LACTB-Flag	This study	
pCLNCX-LACTB-Flag-His	Daniel Bachovchin (Sloan Kettering Institute, New York, USA).	
FUW-LACTB-AAPA + TVGA-Flag-His	This study	
FUW-LACTB-S164A	This study	
pCLNCX-LACTB-S164A-Flag-His	This study	
pLV-shMRPS34-EGFP-blast	VectorBuilder	VB900084-4794rbw
pLV-TRE-MRPS34-blast	VectorBuilder	NM_001300900.2
**Antibodies**		
Antibodies		Table 4
**Oligonucleotides and other sequence-based reagents**		
Primers	This study	Table 2
**Chemicals, enzymes and other reagents**		
Probes	This study	Table 3
MG132	Sigma-Aldrich	M8699
Quick Change XL Site-Directed Mutagenesis Kit	Agilent Technologies	
Recombinant MRPS34 protein	OriGene	TP300834
Granzyme B inhibitor (Z-AAD-CMK)	Sigma-Aldrich	368050
Dde Biotin-PEG4-NHS ester	Click Chemistry Tools	
Dynabeads M-280 Streptavidin	Invitrogen	
MitoTracker Deep Red	Thermo Fisher Scientific	M22426
Duolink PLA kit	Sigma-Aldrich	
TissueArray breast tissue array	TissueArray	BR1191
**Software**		
GraphPad Prism 8	https://www.graphpad.com/	
FlowJo 10.7.1	https://www.flowjo.com/	
ImageJ	https://imagej.net/ij/	
**Other**		

### Cell culture and reagents

Cells were cultivated at 37 °C in a 5% CO_2_-humidified atmosphere. MCF7 cells were purchased from Merck and modified by the introduction of H-RAS V12 oncogene to form estrogen-independent MCF7ras (MCF7R) cells. HMEC cells were purchased from Lonza and immortalized by introducing H-TERT to form HME cells. HME cells were grown in a 1:1 mixture of DMEM/Nutrient Mixture F-12 Ham (Sigma-Aldrich) and Mammary Epithelial Cell Growth Basal Medium (MEBM, Lonza) supplemented with Mammary Epithelial Cell Growth Medium BulletKit™ (MEGM™, Lonza). Cell lines, growth medium, and sources are described in Table 1. All cell lines used in this study were verified to be mycoplasma negative. MG132 was purchased from Sigma-Aldrich (M8699) and used at a final concentration of 20 µM.

**Table 1:** Cell lines, growth medium and sources.

Cell line	Media	Source	RRIDs
HEK293T	DMEM + 10% FBS + 1% P/S	ATCC	CVCL_0063
MCF7/R	DMEM + 10% FBS + 1% P/S	Merck	CVCL_0031
HMEC/HME	MEGM (Lonza)	Lonza	CVCL_3383
HCC1806	RPMI + 10% FBS + 1% P/S	ATCC	CVCL_1258
HCC38	RPMI + 10% FBS + 1% P/S	ATCC	CVCL_1267
HS578T	RPMI + 10% FBS + 1% P/S	ATCC	CVCL_0332
T47D	RPMI + 10% FBS + 1% P/S	ATCC	CVCL_0553
SKBR3	DMEM + 10% IFS + 1%P/S	ATCC	CVCL_0033
CAMA1	DMEM + 10% IFS + 1% P/S	ATCC	CVCL_1115
BT474	RPMI + 10% FBS + 1% P/S	ATCC	CVCL_0179

### Constructs

FUW-LACTB-Flag was derived from FUW-LACTB vector (a kind gift of Dr. Robert Weinberg, MIT) with the introduction of Flag-His tags to the C-terminus of LACTB. pCLNCX-LACTB-Flag-His was a kind gift from Daniel Bachovchin (Sloan Kettering Institute, New York, USA). FUW-LACTB-AAPA + TVGA-Flag-His was generated by mutating the aspartic acids into alanine in the LACTB amino acid residues 73 and 406. FUW-LACTB-S164A and pCLNCX-LACTB-S164A-Flag-His were prepared by mutating Ser164 to Ala164. Mutagenesis was performed using the Quick Change XL Site-Directed Mutagenesis Kit (Agilent Technologies), according to the manufacturer’s protocol. pLV-shMRPS34-EGFP-blast (vector ID: VB900084-4794rbw) and doxycycline-inducible pLV-TRE-MRPS34-blast (NM_001300900.2) were purchased from VectorBuilder. Human MRPS34 cDNA was purchased from VectorBuilder (hMRPS34 [NM_001300900.2]) and sub-cloned into PLVX-puro vector through restriction enzymes BamHI and EcoR1, and a Flag-His-tag was inserted at the MRPS34 C-terminus. The primer sequences used for mutagenesis, cloning, and sequencing are described in Table 2.

**Table 2:** Constructs and primers used for cloning, mutagenesis and/or sequencing.

	Primer	Forward	Reverse
**Mutagenesis**	LACTB-AAPA	GTCCCCCGCGGCCCCCGcCCCTGAGGCGTCGC CTC	GAGGCGACGCCTCAGGGgCGGGGGCCGCG GGGGAC
	LACTB-TVGA	GATTTCTGTCTACAGTGGGTGcCCTTCTGAAAT TTGGG	CCCAAATTCAGAAGGgCACCCACTGTAGA CAGAAATC
	LACTB-S164A	GTTATGCGAATTGCTGCCATCAGCAAAAGTCT C	GAGACTTTTGCTGATGGCAGCAATTCGCAT AAC
**Sub-cloning**	pLVX-MRPS34-flag-His	GATCCTCGAGATGTCTTCAAACTTAATCAGATT TGTTGGAAATCATGATATTGCATCGGAAGGAA AGTTT	GATCGGATCCTAACATCATCATCATCATCAT GACTACAAGGACGACGACGACAAGTTTCGG TGGTTGCT
**Sequencing**	FUW	GGGGAAAGAATAGTAGACATAATAG	TTAGGGGCGGGATGGGCGGAGTTAG
	LACTB 650 -		AGTAATCTTGTTGTGACAGAAACCT
	LACTB 390 +	GTGGTTGGAGTTTCTGTAGATGGAA	
	LACTB 900 +	AAACCTGGTAGTCAGTTTTTGTATT	
	LACTB 1200 +	TTTCTGTCTACAGTGGGTGACCTTC	
	pLVX	AGCTCGTTTAGTGAACCGTCAGATC	CAGCGGGGCTGCTAAAGCGCATGC
**qPCR**	LACTB	CGGGCATAGTGGTTGGAGTTT	TCCCACAATTTGGCAAGAGC
	MRPS34	TGACCTTCAAAGACGCCTCT	CGGTGCTTGTGTCTCCATTT
	GAPDH	CGTATTGGGCGCCTGGTCAC	ATGATGACCCTTTTGGCTCC

The doxycycline-inducible system used throughout this study consists of two separate vectors: one vector carrying the reverse tetracycline-controlled transactivator (rtTA), and the second vector carrying the Tet-responsive element followed by the cloned gene of interest. For activation of the tetracycline-inducible gene expression, the cells were treated with 1 μg/ml of doxycycline hyclate (DOX, Sigma-Aldrich) in the medium. To avoid nonspecific DOX effects, control cells were also treated with DOX, where applicable.

### Lentiviral vector preparation and transduction

About 40% confluent HEK293T cells were transfected with 1 μg of pMDG, 1 μg of pCMV delta R8.2 (Addgene plasmid # 12263), and 1.5 μg of the gene-encoding vector (such as PLVX-puro, FUW-puro, or pLV-blast) in DMEM 10% FBS without antibiotics using X-treme gene HP DNA transfection reagent (Roche) mixed in Opti-MEM (Gibco). Medium in transfected cells was changed after 24 h, and lentiviral vector particles were collected at 48- and 72-h post-transfection. Viral particles were filtered using a 0.45-μm filter.

For lentiviral transduction, cells were seeded at 30% confluency in a 10-cm dish and transduced 24 h later with virus in the presence of 5 μg/ml Polybrene (Merck). Cells were then selected by the relevant selection marker.

### Cell proliferation assay

For cell proliferation assessment, 3.0 × 10^5^ cells were seeded in a 10-cm plate, unless stated otherwise. Twenty-four hours later, after cell attachment, they were treated with 1 μg/ml of doxycycline hyclate (DOX, Sigma-Aldrich). Live cells were counted after 3, 6, 9, and 12 days of DOX treatment using an automatic cell counter (Nano EnTek) with a 1:1 ratio of Trypan blue and medium containing trypsinized and well-homogenized cells. Fresh DOX was added every 3 days.

### Recombinant protein preparation

HEK293T cells expressing pCLNCX-LACTB-Flag-His were grown to 90% confluency in 20 15-cm plates. Cells were washed twice with PBS and scraped. Cell pellets were then collected by centrifugation at 3000 × *g* for 5 min. The pellets were resuspended in cell lysis buffer containing 20 mM TRIS, 150 mM NaCl, 1 mM EDTA, 1 mM EGTA, 1% Igepal, 1% sodium deoxycholate; pH 7.5; debris was removed by centrifugation at 12,000 × *g* for 15 min. LACTB was then captured by incubation of the cell lysate with anti-Flag M2 Magnetic Beads (Sigma). Protein lysate and beads were rotated for 2 h or overnight at 4 °C. Afterward, beads were washed three times with PBS, and 150 µg of 3x Flag Peptide (Sigma) was added to the beads for 1 h at 4 °C, for protein elution. Beads were removed, and the supernatant containing the recombinant protein was concentrated using an Amicon Ultra Centrifugal Filter with a 30 kDa cut-off (Millipore). The final concentration of recombinant protein was determined by a NanoDrop microvolume spectrophotometer. LACTB-purified proteins were kept at 4 °C until used in the experiment. Before subsequent experiments, the quality of the LACTB recombinant protein was validated by an enzymatic cleavage assay to ensure its functional catalytic activity. When the catalytic activity of isolated LACTB was inadequate, the recombinant protein was discarded. Recombinant proteins for MRPS34 (TP300834), GAPDH (TP302309), COQ6 (TP760736), HSBP1 (TP301399), ASCL3 (TP320042), and TOMM20 (TP310746) were purchased from OriGene. Recombinant HIGD1A protein (ab125988) was purchased from Abcam. Proteins were aliquoted and stored at −80 °C.

### LACTB in vitro substrate assay

About 150 ng of recombinant protein was added to a Nunc MicroWell 96-well black, clear optical-bottom plate with polymer base (Sigma-Aldrich) in PBS, together with 1 µl of peptide substrate Ac/nonAc-xxxD-AMC (Enzo Life Sciences and IOCB peptide synthesis facility) (prepared in DMSO at 10 mM) at a final volume of 100 μl. Where needed, experiments were performed with Granzyme B inhibitor (Sigma-Aldrich, 368050), or PBS was substituted with H_2_O. Fluorescence was measured on a Spark Multimode Plate Reader (Tecan) for 50- to 180-min intervals at 25 °C, with a kinetic cycle of 10 min, fluorescent top reading, multiple reads per well, and an excitation wavelength of 380 nm and an emission wavelength of 460 nm. The peptide substrates used contained aspartic acids present within the LACTB sequence (Table 3); as a negative control, we used a tetrapeptide ending in alanine instead of aspartic acid: Ac-YGAA-AMC; as a positive control, we used a tetrapeptide that has already been described to be cleaved by LACTB: Ac-YVAD-AMC (Keckesova et al, 2017).

**Table 3:** Aspartic acid - containing peptide sequences present in the LACTB.

Probes (Ac/nonAc) used for testing enzymatic activity of LACTB
AAPD-AMC
SSRD-AMC
RIKD-AMC
VSVD-AMC
GYAD-AMC
GKLD-AMC
LDLD-AMC
YEKD-AMC
EKND-AMC
KKND-AMC
FKND-AMC
KWLD-AMC
IFHD-AMC
HDLD-AMC
PYVD-AMC
TVGD-AMC
MSWD-AMC
EELD-AMC
LEFD-AMC
FDKD-AMC
DRSD-AMC
YGAA-AMC

### LACTB autoproteolytic activity assay

Purified recombinant LACTB in H_2_O (distilled and autoclaved) or PBS was incubated at 25 °C, with and without Z-AAD-CMK (Granzyme B inhibitor), which was already described to inhibit LACTB activity [30], for different time points. Samples were then processed for Western Blot analysis and immunoblotted for LACTB (as described below). Of note, the timing of the onset of autoproteolytic activity depends on the quality and purity of the recombinant LACTB and on the duration of storage before the experiment. Therefore, based on these parameters, it sometimes manifests several hours earlier or later than the average times shown in this manuscript.

### Modified PICS assay

Mitochondria were isolated from twelve 15-cm plates of confluent MCF7R cells. Mitochondria isolation was done using Qproteome Mitochondria Isolation Kit (Qiagen), according to the manufacturer’s instructions. Isolated mitochondria were dissolved in 1 ml of Outer Mitochondrial Membrane Disruption Buffer (10 mM HEPES and 1 mM EDTA, pH 7.4) and incubated for 30 min at 4 °C. Then 50 µl of 3 M KCl (stock concentration) was added and incubated for 10 min at 4 °C. After 10 min sample was spin down at 11,000×*g* for 10 min, the supernatant was sonicated, and the pellet was dissolved in 1 ml of Inner Mitochondrial Membrane Disruption Buffer (5 mM HEPES, 5 mM sucrose and 1 mM EGTA, pH 7.2), incubated for 30 min at 4 °C and then sonicated. To both sonicated samples, RQ1 DNase (Promega) was added for 30 min at 25 °C. The mixture was then centrifuged at 11,000×*g*; the pellets were kept for later use, and the supernatant was concentrated to a final volume of 200 µl using an Amicon filter with a 3 kDa cut-off (Millipore). Protein concentration in the sample was then determined using the DC Protein Assay (Bio-Rad). Sulfate groups were blocked using 5 mM of dithiothreitol (DTT) that was added to the isolated mitochondrial proteome sample and incubated at 37 °C for 1 h. This was followed by the addition of 40 mM iodoacetamide and incubation at 37 °C for 1.5 h. The reaction was terminated by adding another 15 mM DTT and incubating for 10 min at 37 °C. Afterward, amino groups were blocked with 60 mM formaldehyde and 60 mM sodium cyanoborohydride for 18 h at 37 °C. The reaction was stopped by the addition of 35 mM glycine. The final sample was washed 15 times with PBS using an Amicon filter with a 3 kDa cut-off (Millipore) to reduce glycine concentration. Recombinant LACTB-WT or LACTB-S164A was added to the sample of mitochondrial proteins with blocked reactive groups. The addition was done in two steps: first, 0.5 µg of LACTB was added, and the mixture was incubated for 2 h at 25 °C. Then another 0.5 µg of LACTB was added, and the mixture was incubated for 2 h at 37 °C. To trap newly formed primary amines, the sample was incubated with 0.5 µl of a 10 mM solution of Dde Biotin-PEG4-NHS ester (Click Chemistry Tools) in DMSO for 2 h at 25 °C. Biotinylated proteins were then purified using Streptavidin Magnetic Beads (Dynabeads™ M-280 Streptavidin, Invitrogen) and incubated at 25 °C for 30 min with mild agitation. Beads were then collected, washed thoroughly with 1 ml of different solutions in the following order—PBS, 1 M guanidin HCl, PBS, 20% isopropanol, PBS, 5% DMSO, PBS, 5% ACN, and ten times beads equilibration buffer (50 mM HEPES and 150 mM NaCl, pH 7.5). Bound proteins were then eluted using 2% hydrazine in 100 mM sodium phosphate and analysed by mass spectrometry.

### Immunoprecipitation (IP) assay

IP was done with crosslinking and non-crosslinking conditions. MCF7R-RTTA-hygro-FUW-LACTB-WT (MCF7R-LACTB-WT) cells were treated with DOX for 1 day and then washed twice with PBS. For crosslinking conditions: 1 mM DSP (Thermo Scientific) in PBS was added to the cells for 20 min at room temperature, followed by 10 mM Tris, pH 7.5, for 15 min at room temperature to stop the DSP reaction. Cells were lysed in IP-lysis buffer containing 20 mM Tris, 150 mM NaCl, 1 mM EDTA, 1 mM EGTA, 0.5% Triton X-100, and supplemented with protease (cOmplete, Roche Diagnostics) and phosphatase (PhoStop, Roche Diagnostics) inhibitors. Cell lysates were sonicated three times for 10 s and centrifuged at 12,000×*g* for 15 min to remove cell debris. Protein lysates were quantified using the DC protein assay kit (Bio-Rad). About 2 μg of antibody was added to 50 μl of protein G magnetic Dynabeads (50% slurry) previously washed in IP-lysis buffer. Beads with the antibody were rotating for 2 h at 4 °C. For crosslinking conditions, 20 mM DMP (Thermo Scientific) in sodium borate, pH 9, was added to the beads, which were rotated for 30 min at 25 °C. The process was repeated twice; 50 mM glycine, pH 2.5, was used to stop the DMP reaction. About 1.5 mg of protein lysate was added to the beads, which had been prewashed with IP-lysis buffer. Lysates with beads were rotating for at least 2 h at 4 °C. Protein was eluted from the beads by boiling for 10 min at 98 °C in Laemmli buffer (Bio-Rad) containing a reducing agent (Invitrogen). Co-IP for LC-MS/MS analysis: beads were rinsed 3x with 20 mM Tris, pH 8.0, to remove salts and detergent, and kept in this buffer until the analysis. Bound proteins (LACTB and co-immunoprecipitated proteins) were analyzed via LC-MS/MS after in-solution trypsin digestion. Beads with no loaded antibody were used as a control for nonspecific interactions. Each condition was prepared in triplicate. Proteins detected in LACTB co-IP replicates and undetected in the control sample were considered as positive hits.

### Quantitative RT-PCR

Total RNA was isolated directly from cultured cells using the RNeasy Plus Mini kit (Qiagen), following the manufacturer’s instructions. Reverse transcription was performed with a High-Capacity cDNA Reverse Transcription Kit (Life Technologies). mRNA levels were measured with gene-specific primers using the SYBR Green I master mix (Roche) in a Roche LightCycler 480 system (Roche) with a 384-well plate; each well contained 25 ng/ml cDNA. The amplification program consisted of an initial cycle of 95 °C for 15 s, denaturation at 95 °C, followed by 45 cycles of 60 °C. After amplification, quantification cycle (Cq) values were reported by the Roche LightCycler 480 system. Cq values for each sample were normalized to the geometric mean Cq value of the reference gene, DCT. Then, the DDCT was calculated, which refers to the Cq of the sample of interest. Lastly, the value of 2-DDCt was considered. The obtained data were then further normalized to the expression of these genes relative to the control cells. GAPDH was used as a reference gene for normalization. The PCR primer sequences are listed in Table 2.

### Western blot analysis

Proteins were extracted from cells using RIPA buffer (Sigma) supplemented with protease and phosphatase inhibitors. Approximately 20 μg of protein lysate was loaded into SDS-PAGE 4% to 15% gradient gels (Bio-Rad). Western blots and transfers were performed following standard protocols using a wet-transfer system (Bio-Rad) onto PVDF membranes (Immobilon-P, Millipore). Next, membranes were blocked for 1 h with 5% non-fat milk in PBS containing 0.1% Tween-20 (v/v), followed by overnight blotting with relevant antibodies at 4 °C. Horseradish peroxidase-conjugated secondary antibodies were used (Cell Signaling, 1:5000). Blots were developed using enhanced chemiluminescence (Dura or Femto, Thermo Scientific), and the signal was detected using Azure c600 Western blot Imaging system. Antibodies used are described in Table 4.

**Table 4:** Antibodies and reagents used in the study.

Antibodies and reagents	Dilution or concentration	Catalog #
ATP5A	WB: 1000X	AB14748
beta-actin	WB: 1000X	66009-1-lg
DAP3	WB: 1000X	10276-1-AP
GAPDH	WB: 1000X	60004-1-lg
HSP90	WB: 3000X	13171-1-AP
LACTB-rabbit	IHC: 250X	PAJ512Hu01
LACTB-rabbit	WB: 1000X, IF: 250X	18195-1-AP
Mitochondria, surface of intact mitochondria clone 113-1	IF: 250X	MAB1273
MRPS22	WB: 1000X	10984-1-AP
MRPS34-rabbit	WB: 500X, IF: 200X, IHC: 250X	15166-1-AP
MitoTracker Deep Red	IF: 0.25 µM	M22426

### Mass spectrometry stoichiometry analysis

Protein samples were prepared using the filter-aided sample preparation (FASP) method, including buffer exchange, reduction/alkylation, and proteolytic digestion to peptides. The resulting peptides were analyzed by nanoLC–MS/MS using an UltiMate 3000 system coupled to an Orbitrap Fusion Lumos mass spectrometer (Thermo Fisher Scientific). Peptides were separated on a 50 cm EASY-Spray C18 column (Thermo Fisher Scientific) and acquired in DIA mode. Raw data were processed in Spectronaut for protein identification and quantification, and iBAQ values were used for downstream analysis. For each sample, the MRPS34/LACTB iBAQ ratio was calculated and compared between the HME and MCF7ras groups.

### Dot blot analysis

PVDF membranes were activated in methanol and placed on the top of a marked grid of Whatman filter paper soaked in transblotting buffer. Recombinant proteins were spotted onto the wet membrane and incubated for 15 min to fix the proteins. The membrane was then blocked for 1 h in 5% non-fat milk in PBS with 0.1% Tween-20 (v/v), followed by a 2-h incubation at room temperature with recombinant LACTB (around 10 μg) in PBS. The membrane was washed three times in PBS-T and blotted overnight at 4 °C using LACTB antibody.

### In vivo mouse experiments

All manipulations with cells and mice were performed in Class II Biological Safety Cabinets to maintain an aseptic working environment throughout the study. Cells were cultivated in media (DMEM) + 10% FBS + 1% Penicillin-streptomycin and suspended in 50% Matrigel prior to injections. Adult female NSG (NOD.Cg-Prkdcscid Il2rgtm1Wjl/SzJ) mice were obtained from The Jackson Laboratory. The injected numbers of MCF7R cells were 3 × 10^5^, and of HCC1806 were 1.5 × 10^5^, prepared in 15–30 μL for each dose, applied bilaterally into the mammary fat pad. Body condition score and tumor size were measured twice weekly. The in vivo DOX treatment was administered via drinking water containing 2 mg/mL DOX and 10 mg/mL sucrose when tumors reached approximately 4 mm in diameter. The mice were euthanized when the tumors reached a cumulative size of 1.5 cm or earlier. Sample sizes were chosen to reach statistical significance, and tumor measurements and data analysis were performed in a blinded fashion. The animal experiment was approved by the Animal Research Ethics Committee of the Czech Academy of Sciences (approval ID: AVCR 5282/2021 SOV II).

### Immunofluorescence (IF) assay

LACTB-WT and LACTB-AAPA + TVGA overexpression was induced in MCF7R cells for 1 day, in 13 mm glass coverslips. Twenty-four hours after, cells were washed two times in PBS for 4 min and subsequently fixed with 4% paraformaldehyde (PFA) and permeabilized with 0.1% Triton X-100 in PBS, for 20 min. Cells were washed three more times with PBS for 10 min each and blocked with 5% BSA in PBS for 30 min. After this step, the coverslips were transferred to a wet chamber, where 50 μl of the diluted primary antibody was added to each coverslip for at least 1 h. Afterward, the primary antibody was removed, and cells were washed three times with PBS-0.1% Tween (PBS-T), followed by incubation with Alexa Fluor secondary antibodies for 1 h. Cells were then washed three times with PBS-T, and coverslips were mounted in ProLong Gold anti-fade reagent with DAPI (Life Technologies). All steps were performed at room temperature. Antibodies used are described in Table 4.

Images were acquired on a Zeiss LSM 980 confocal microscope using 405 and 561 nm laser excitation wavelengths with a 63× (NA 1.4) immersion oil objective. Images correspond to a single channel and a single section in the z-axis, in the middle of the cell. Images were analyzed with ImageJ. Antibodies used are described in Table 4.

### Proximity ligation assay (PLA)

To evaluate the interaction between endogenous LACTB and the potential binding partner proteins in situ, we used the Duolink proximity ligation assay (PLA) (Sigma-Aldrich) in MCF7R control and MCF7R-FUW-LACTB-WT following the manufacturer’s instructions. Briefly, cells were grown on eight-well chamber slides (Lab-Tek Chamber Slide, 177445). For mitochondrial staining, cells were incubated with MitoTracker Deep Red (Thermo Fisher, M22426) at a final concentration of 0.25 µM for 40 min at 37 °C. The cells were fixed with prewarmed 4,2% PFA, permeabilized with 0,1% Triton X-100, incubated in blocking solution (provided by the kit) for 1 h at 37 °C, and then incubated overnight in a solution containing the primary antibodies (LACTB and the potential binding partner/control protein). PLA probes were prepared by diluting 1:5 in antibody diluent (provided with the kit), then added to the slides and incubated for 1 h at 37 °C. The ligation of the PLA probes was performed by incubating the cells in the ligation solution for 30 min at 37 °C, followed by a 100 min amplification step at 30 °C. The slides were then allowed to dry in the dark at room temperature and mounted with Duolink In Situ Mounting Medium with DAPI (Sigma-Aldrich). Images were acquired in a confocal microscope (Zeiss) as described above. Quantification of PLA dots was performed as described in (López-Cano et al, 2019).

### Immunohistochemistry (IHC)

Human tumor and normal tissue sections (obtained from TissueArray; BR1191) were deparaffinized by heating the slides at 60 °C for 10 min. Next, the slides were washed twice in xylene for 5 min each, and once in 1:1 xylene:ethanol for 3 min. Then, the slides were hydrated in washes with 100 (twice), 95, 80, 75, and 50% ethanol for 3 min each, followed by washes with 0.85% NaCl, PBS, and H_2_O for 5 min each. Tissues were boiled for 20 min in 1x citrate buffer (Sigma-Aldrich). Slides were washed in PBS for 1 min, and then permeabilized with 0.2% Triton X-100 for 20 min. The blocking solution was added for 20 min (30% horse serum in PBS, 0.1% Tween-20 at room temperature). Sections were incubated overnight at 4 °C with the primary antibody (described in Table 4). After three washes with PBS, sections were incubated with secondary antibodies (VECTASTAIN ABC-AP) for 1 h at room temperature, and then washed three times with PBS. Then, the slides were incubated with Fast Red solution (Abcam). Hematoxylin was used as a counterstain to stain the nucleus. Slides were dehydrated and mounted. IHC images were acquired using an optical light microscope (Nikon).

### EdU staining

MCF7R cells overexpressing LACTB, MRPS34, and LACTB + MRPS34 at different time points were collected. Cells were treated with 20 μM EdU for 90 min at the end of LACTB/MRPS34 induction. About 600,000 cells were collected for all the conditions. Edu labeling was performed for 30 min using 2 mM CuSO4, 8 μM CY-5 (Sigma), and 20 mg/ml ascorbic acid. Cells were also stained with 3 μg/ml DAPI for 15 min to visualize DNA. The signal was detected by flow cytometry, and the data were analyzed using FlowJo 10.7.1.

### Statistical analysis

Data were shown as the average ± standard deviation (SD). Comparisons of quantitative variables following normal distributions were evaluated using Student’s *t*-test for paired or unpaired samples, as appropriate. For in vivo experiments, tumor growth differences were analyzed using two-way ANOVA. Differences were considered significant when the *p* value ≤0.05. Statistical analysis was performed using GraphPad Prism 8 software.

## Supplementary information

Peer Review File

Dataset EV1

Dataset EV2

Dataset EV3

Source data Fig. 1

Source data Fig. 2

Source data Fig. 3

Source data Fig. 4

Source data Fig. 5

Expanded View Figures

## Data Availability

This study includes no data deposited in external repositories. The source data of this paper are collected in the following database record: biostudies:S-SCDT-10_1038-S44319-026-00936-9.
