# Supplementary material for: The role of autoproteolysis and mitoribosomal proteins in the regulation of the mitochondrial LACTB tumor suppressor
Source: EMBO Rep. 2026 Sep 26;27(21):6342–65. doi: 10.1038/s44319-026-00936-9 (PMC13743786; doi:10.1038/s44319-026-00936-9)
Supplement: Supplementary file 1 [file 44319_2026_936_MOESM10_ESM.pdf]

Expanded View Figures

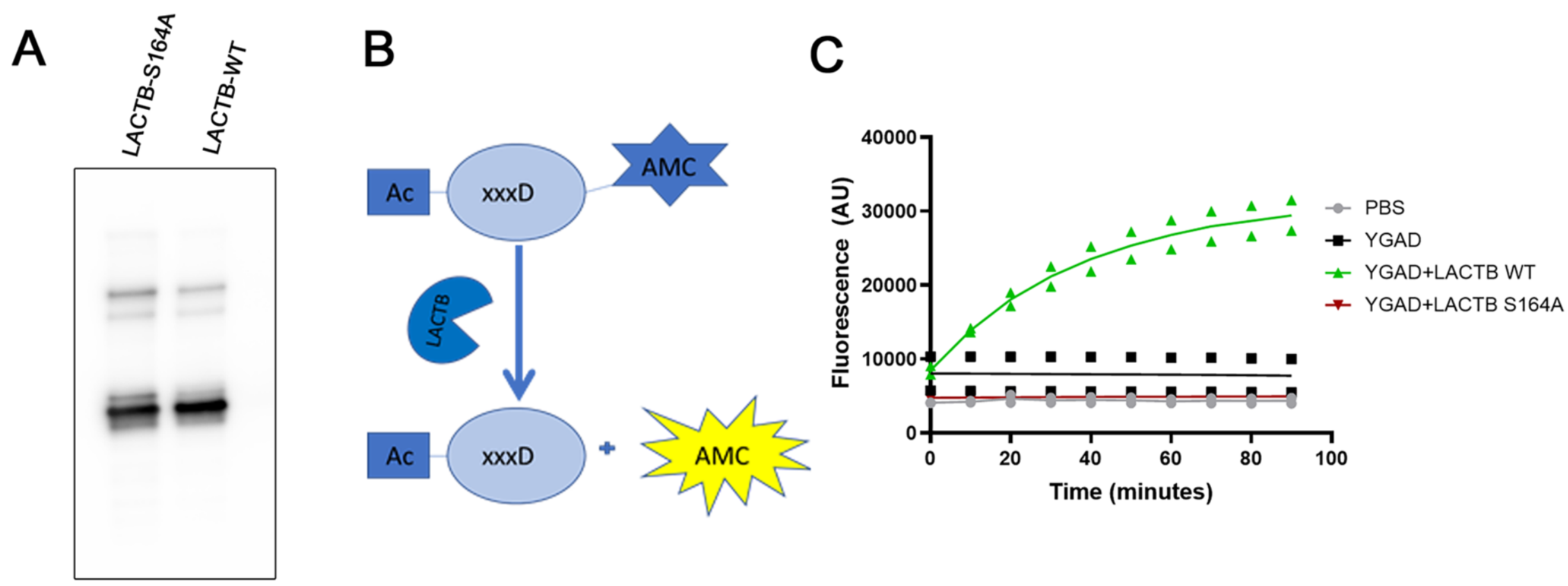

Figure EV1. Expression and activity of LACTB recombinant protein.

(**A**) The expression level of LACTB-WT and LACTB-S164A recombinant proteins is shown by Western blot analysis. (**B**) Graphical illustration of LACTB's enzymatic activity assay using tetrapeptides (such as YVAD and YGAD) linked to AMC fluorescent probe. Upon cleavage of the probe by LACTB, the AMC moiety is released and emits fluorescence. (**C**) Comparison of the cleavage ability of LACTB-WT and LACTB-S164A catalytic mutant against the YGAD probe. The experiment was performed twice ($N = 2$). Mean ± SD.

 © The Author(s)

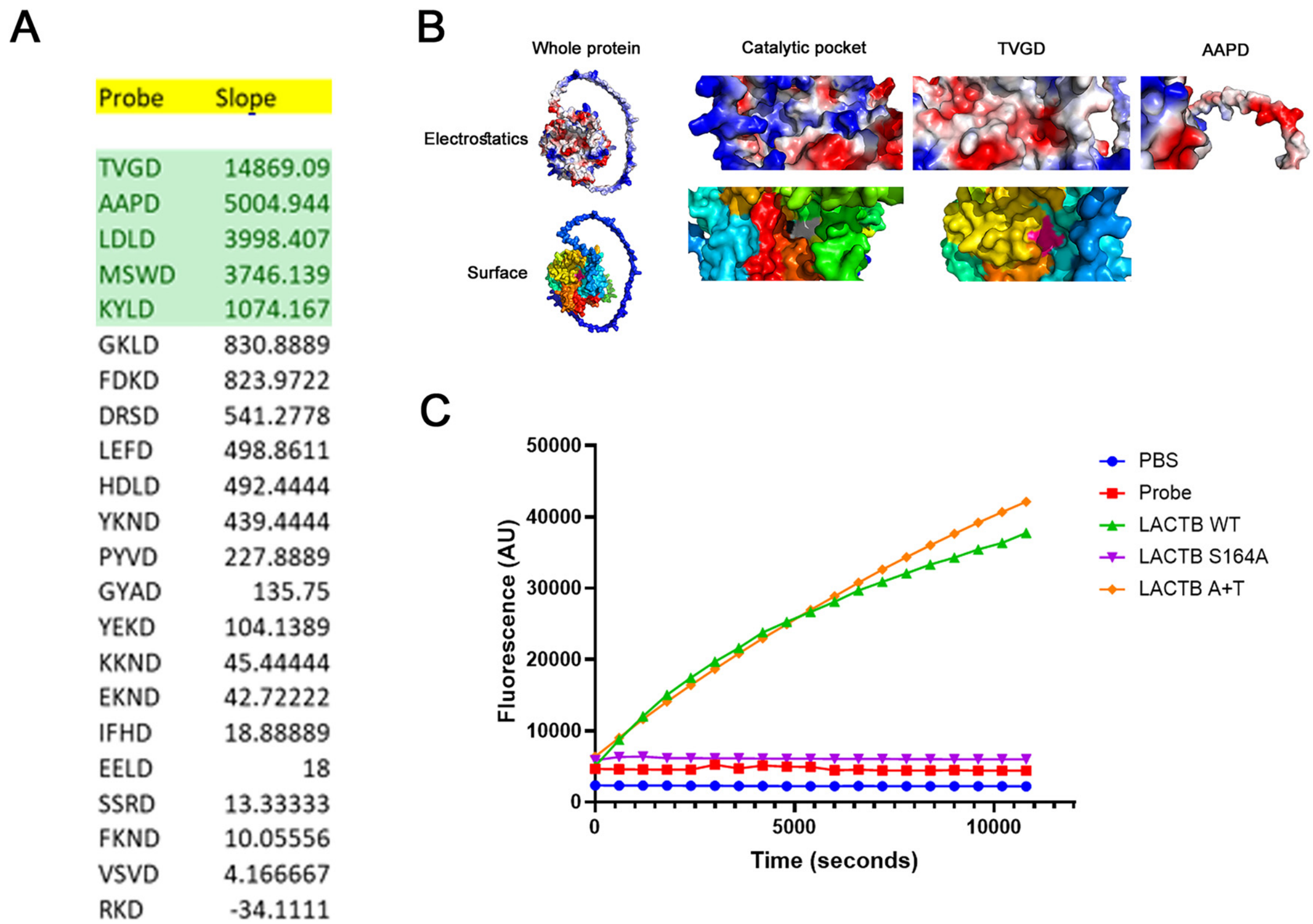

Figure EV2. The role and position of AAPD/TVGD residues in LACTB.

(**A**) The list of peptides, fused to AMC fluorescent probe, representing all tetrapeptides ending in aspartic acid within the LACTB sequence that were tested for their ability to be cleaved by LACTB. Highlighted in the table are the peptides most efficiently cleaved by LACTB, as calculated by subtracting the first fluorescence measurement from the last fluorescence measurement (slope). (**B**) Upper panel: electrostatic comparison between the catalytic pocket and TVGD and AAPD sites; Bottom panel: surface analysis of full-length LACTB-WT, showing the catalytic pocket (gray) and TVGD (magenta) regions. (**C**) Enzymatic activity of the autoproteolytic mutant LACTB-AAPA + TVGA (A + T) compared to the catalytically inactive mutant S164A and LACTB-WT. The experiment was performed twice ($N = 2$).

© The Author(s)

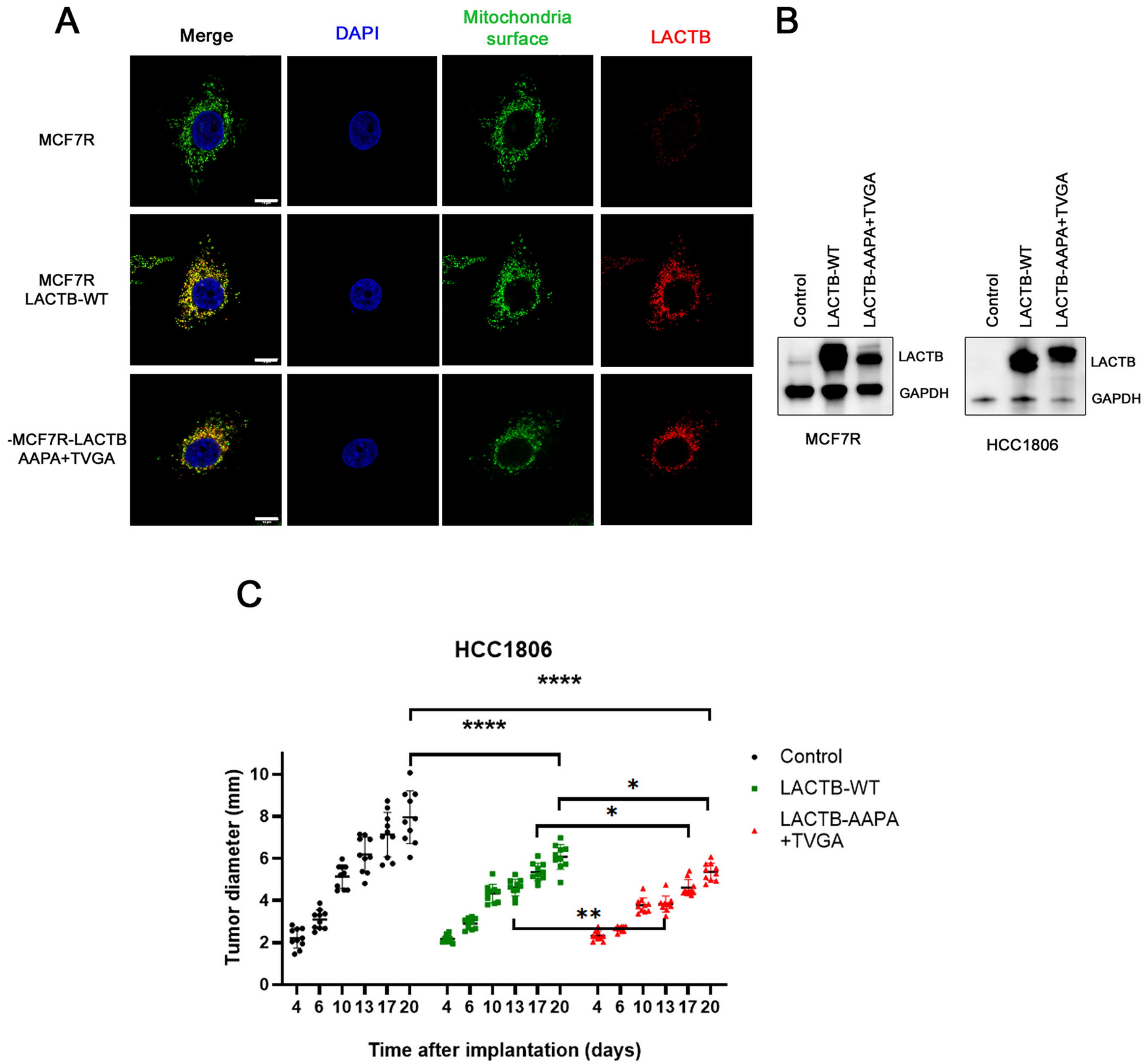

Figure EV3. Expression and effect of LACTB-AAPA+TVGA in cells and tumors.

(**A**) Confocal microscopy images of the localization of LACTB-WT and LACTB-AAPA + TVGA in MCF7R, after one day of induction. Blue corresponds to DAPI, green labels mitochondria surface (Merck, MAB1273), and red represents LACTB. Scale bar is 5 µm. (**B**) Expression levels of LACTB-WT and LACTB-AAPA + TVGA in MCF7R and HCC1806 cells. (**C**) Growth of tumors formed by injection of control HCC1806 (black), HCC1806 LACTB-WT (green), and HCC1806 LACTB-AAPA + TVGA (red) cells into the mouse mammary fat pad. Doxycycline treatment was initiated at 6 days of tumor growth (blue arrows). Statistics were calculated using two-way ANOVA, $*p < 0.05$; $**p < 0.01$, $****p < 0.0001$. $N = 10$. Mean ± SD. $*p < 0.05$; $**p < 0.01$, $****p < 0.0001$. HCC1806 control vs HCC1806_LACTB at 20 days, $p = 1.72 \times 10^{-11}$; HCC1806 control vs HCC1806_LACTB-AAPA + TVGA at 20 days, $p = 7.3 \times 10^{-19}$; HCC1806_LACTB vs HCC1806_LACTB-AAPA + TVGA at 20 days, $p = 0.0147$; HCC1806_LACTB vs HCC1806_LACTB-AAPA + TVGA at 17 days, $p = 0.0105$; HCC1806_LACTB vs HCC1806_LACTB-AAPA + TVGA at 13 days, $p = 0.0065$.

 © The Author(s)

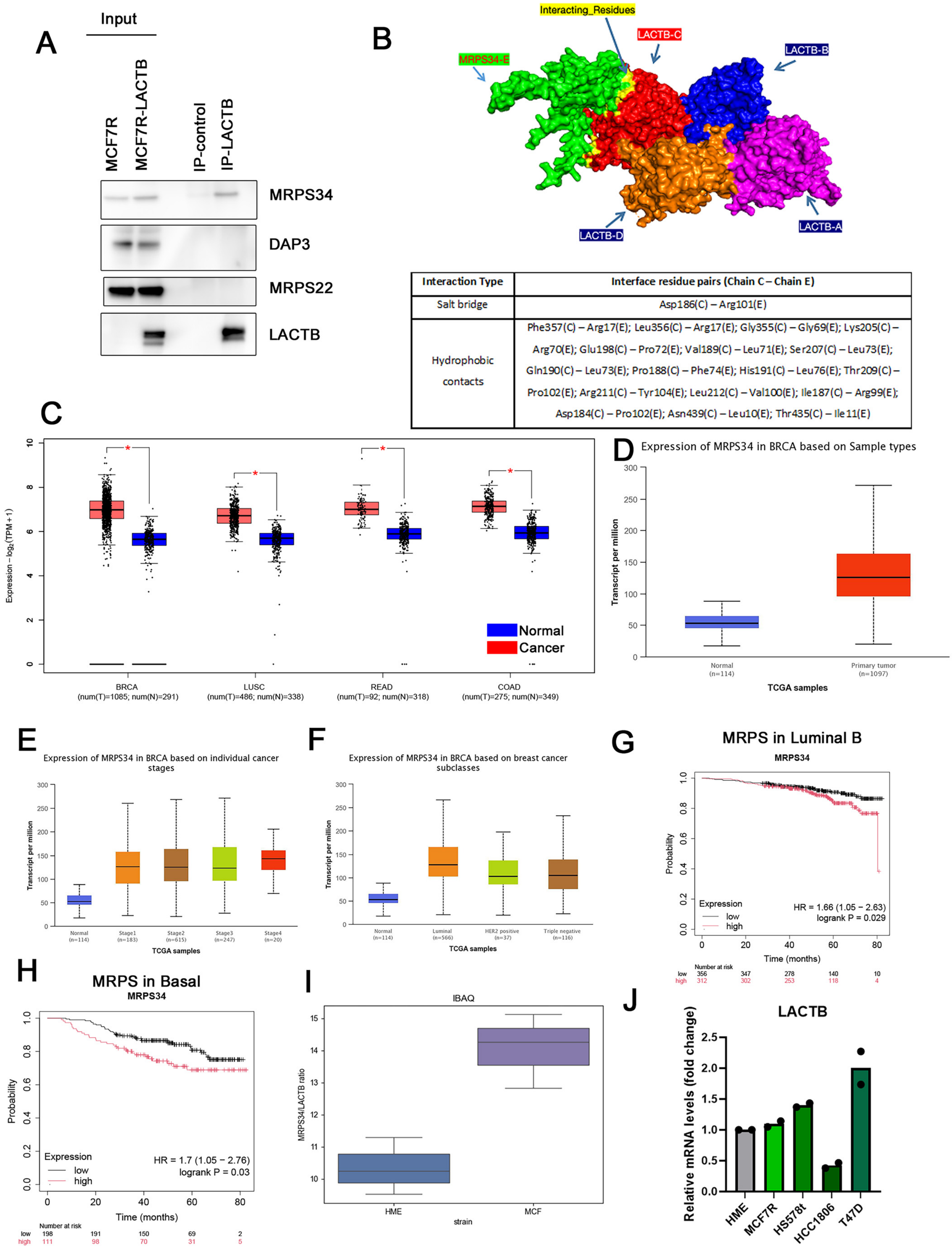

Interaction Type	Interface residue pairs (Chain C – Chain E)
Salt bridge	Asp186(C) – Arg101(E)
Hydrophobic contacts	Phe357(C) – Arg17(E); Leu356(C) – Arg17(E); Gly355(C) – Gly69(E); Lys205(C) – Arg70(E); Glu198(C) – Pro72(E); Val189(C) – Leu71(E); Ser207(C) – Leu73(E); Gln190(C) – Leu73(E); Pro188(C) – Phe74(E); His191(C) – Leu76(E); Thr209(C) – Pro102(E); Arg211(C) – Tyr104(E); Leu212(C) – Val100(E); Ile187(C) – Arg99(E); Asp184(C) – Pro102(E); Asn439(C) – Leu10(E); Thr435(C) – Ile11(E)

© The Author(s)

Figure EV4. Association between MRPS34 and LACTB.

(**A**) Immunoprecipitation (IP) through LACTB antibody followed by Western blot with antibodies against MRPS34, DAP3/MRPS29, and MRPS22. The input consists of lysate of MCF7R and MCF7R overexpressing doxycycline-inducible LACTB for 1 day. IP-control was done by immunoprecipitating with Resistin IgG antibody. Part of this figure is shared with Fig. 4A. (**B**) Structural depiction of the interaction between the LACTB tetramer and MRPS34. The LACTB subunits (chains A–D) are illustrated in magenta, blue, orange, and red, respectively, while MRPS34 (chain E) is shown in green. Residues involved in interfacial contacts are highlighted in yellow. The accompanying table details the interface interactions, categorizing residue pairs into salt bridges and hydrophobic contacts. (**C**) Expression of MRPS34 in normal (blue) and cancer (red) tissues of breast (cancer $N = 1085$; normal $N = 291$, $p = 1.62 \times 10^{-12}$), lung (cancer $N = 486$; normal $N = 338$, $p = 1.62 \times 10^{-12}$), rectum (cancer $N = 92$; normal $N = 318$, $p = 1.64 \times 10^{-12}$), and colon cancer (cancer $N = 275$; normal $N = 349$, $p = 1.62 \times 10^{-12}$). The graph was obtained using the GEPIA2 tool. Box plots show the median (center line), 25th–75th percentiles (box bounds), and whiskers indicate the minimum and maximum values. (**D–F**) Expression of MRPS34 in normal and breast cancer (BRCA) tissues stratified by different stages and subtypes. Graph obtained using the UALCAN tool. In panel (**D**), normal $N = 114$, primary tumor $N = 1097$; in panel (**E**), normal $N = 114$, stage 1 $N = 183$, stage 2 $N = 615$, stage 3 $N = 247$ and stage 4 $N = 20$; in panel (**F**), normal $N = 114$. Luminal $N = 566$, HER2 positive $N = 37$ and triple negative $N = 116$. (**G**) Effects of the expression of MRPS34 on overall survival in patients with luminal B breast cancer, using the KMplotter tool. (**H**) Effects of MRPS34 expression on overall survival in patients with basal breast cancer, using the KMplotter tool. (**I**) Mass spectrometry IBAQ analysis examining the stoichiometry of MRPS34 and LACTB in non-tumorigenic HME cells and tumorigenic MCF7ras cells. Data of three replicates is shown ($N = 3$). (**J**) RT-PCR analysis of endogenous LACTB levels, normalized to GAPDH, in non-tumorigenic HME cells, and in cancer cell lines MCF7R, HCC1806, T47D and HCC38. Samples were shared with Fig. 4E, where we assessed the MRPS34 levels. Mean ± SD. The experiment was performed twice ($N = 2$).

© The Author(s)

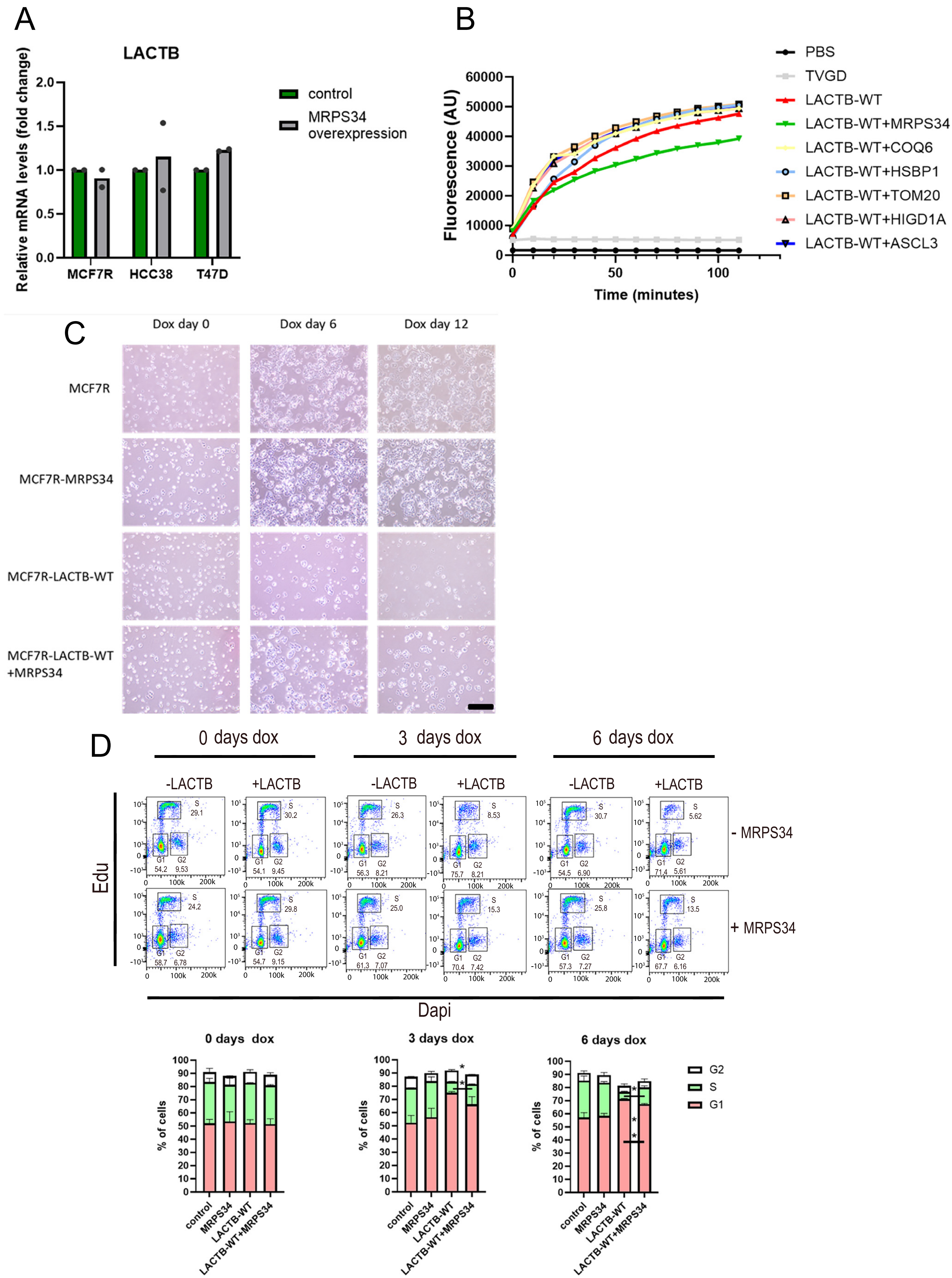
A
LACTB
Relative mRNA levels (fold change)
control
MRPS34 overexpression
MCF7R
HCC38
T47D
B
Fluorescence (AU)
Time (minutes)
PBS
TVGD
LACTB-WT
LACTB-WT+MRPS34
LACTB-WT+COQ6
LACTB-WT+HSBP1
LACTB-WT+TOM20
LACTB-WT+HIGD1A
LACTB-WT+ASCL3
C
Dox day 0
Dox day 6
Dox day 12
MCF7R
MCF7R-MRPS34
MCF7R-LACTB-WT
MCF7R-LACTB-WT +MRPS34
D
0 days dox
3 days dox
6 days dox
-LACTB
+LACTB
- MRPS34
+ MRPS34
Edu
Dapi
% of cells
control
MRPS34
LACTB-WT
LACTB-WT+MRPS34
G2
S
G1

Figure EV5. MRPS34 negatively affects LACTB in cells.

(**A**) Endogenous LACTB mRNA levels in MCF7R, HCC38, and T47D cancer cells with or without MRPS34 induction for 1 day, normalized to GAPDH. The experiment was performed twice ($N = 2$). Mean ± SD (**B**) Enzymatic activity of LACTB directed against a peptide probe in the presence of MRPS34 and other mitochondrial proteins. LACTB-WT was pre-incubated with the respective proteins for 48 h at 25 °C prior to the assessment of its enzymatic activity. (**C**) Light microscopy images of MCF7R cells, expressing LACTB, MRPS34, or both for 0, 6, and 12 days. Scale bar: 500 µm. (**D**) Flow cytometry-based cell cycle analysis of EdU/DAPI in MCF7R cells. Comparison of the DNA synthesis (S phase) and G1 phase in MCF7R-LACTB-WT and MCF7R-LACTB-WT + MRPS34 cells upon doxycycline induction (day 0, 3, and 6), using Student's *t*-test. Experiments were performed three times ($N = 3$). Significance was reached when $P < 0.05$. *$p < 0.05$; **$p < 0.01$. Mean ± SD represented. *$p < 0.05$; **$p < 0.01$. MCF7R-LACTB-WT vs MCF7R MRPS34 3 days dox, $p = 0.0022$; S phase of MCF7R-LACTB-WT vs MCF7R MRPS34 6 days dox, $p = 0.0187$; G1 phase of MCF7R-LACTB-WT vs MCF7R MRPS34 6 days dox, $p = 0.0040$.

 © The Author(s)
