# Supplementary material for: The role of autoproteolysis and mitoribosomal proteins in the regulation of the mitochondrial LACTB tumor suppressor
Source: EMBO Rep. 2026 Sep 26;27(21):6342–65. doi: 10.1038/s44319-026-00936-9 (PMC13743786; doi:10.1038/s44319-026-00936-9)
Supplement: Supplementary file 2 [file 44319_2026_936_MOESM1_ESM.pdf]

EMBO reports

# The role of autoproteolysis and mitoribosomal proteins in the regulation of LACTB tumor suppressor

Sara Escudeiro Lopes, Juan Gonzalez-Morena, Alzbeta Baudysova, Michal Svoboda, Pavel Marášek, Gabriel Rodriguez-Gomez, Jessica Ferreira Mendes, Chung Weng Phang, Sravan Kumar Miryala, Pavel Jakoube, Beata Kovarova, Susana Machado, Yuxiong Feng, and Zuzana Keckesova

*Corresponding author(s): Zuzana Keckesova (keckesova@uochb.cas.cz)*



## Review Timeline:

| | |
|---|---|
| Submission Date: | 11th Jan 26 |
| Editorial Decision: | 28th Jan 26 |
| Revision Received: | 16th Mar 26 |
| Editorial Decision: | 3rd Apr 26 |
| Revision Received: | 12th May 26 |
| Editorial Decision: | 6th Aug 26 |
| Revision Received: | 11th Aug 26 |
| Accepted: | 12th Aug 26 |

*Editor: Bernd Pulverer*

## Transaction Report:

(Note: With the exception of the correction of typographical or spelling errors that could be a source of ambiguity, letters and reports are not edited. Depending on transfer agreements, referee reports obtained elsewhere may or may not be included in this compilation. Referee reports are anonymous unless the Referee chooses to sign their reports.)

# Point-by-Point Response to Reviewers

We thank the reviewers for insightful and constructive comments. We are grateful for the opportunity to revise our manuscript and to answer their concerns and questions through additional experiments, discussion, and clarifications.

## Reviewer #1:

We thank the reviewer for the thoughtful comments and for recognizing the extensive work and data presented in support of our conclusions. We appreciate the opportunity to address the reviewer's concerns through the following experimental additions and discussion.
Reviewer's comments are highlighted in grey.

**Major point/General Comment:**

**1.** Data for the relative level of LACTB and MRPS34 in breast cancer patients, relative to tumor status or response to therapy, is lacking. Such data is crucial to support the relevance of the authors' findings. Differences observed upon manipulation of LACTB or MRPS34 or PNH733 require notable time (30h or more) and, in most cases, are not compelling (see below), raising the concern that other key players are as if not more important.

**Response:** These points were experimentally addressed and answers are provided below. Specifically, the LACTB and MRPS34 expression in breast cancer patients is explained in **Point 3** and the differences observed at extended manipulation of LACTB and MRPS34 are explained in **Point 4**.

**Other comments/Minor points:**

**1.** Fig 1 – Authors decide to focus on LACTB, yet other hits were identified in their initial analysis (panel B) – why were they ignored? What are the different bands shown (panel C)? how was this assay performed? Here and elsewhere, details of experiments are lacking. Is this a western? What antibodies were used?

**Response:** The other hits were not ignored; we are actively investigating them. However, from the beginning, we focused our attention on LACTB because it was one of the most compelling hits, and the idea that LACTB might be cleaving itself was particularly fascinating.
In scientific research, once a list of candidate proteins is identified through rigorous and established criteria such as multiple biological replicates, p-values, and fold-changes, the final selection for in-depth validation is left to the discretion of the researcher. There is no requirement, either scientifically or from the publishing community, to test all identified hits within a single manuscript. In fact, doing so would be impractical in terms of space, funding, and clarity. Our decision to focus on LACTB does not diminish the significance of the discoveries presented in our manuscript. The findings stand on their own, contributing valuable insights to this line of research.

**Panel C** depicts recombinant LACTB protein, which was obtained by pulling down, purifying, and concentrating LACTB expressed in mammalian HEK293T cells. The protein was visualized via Western blot using a polyclonal LACTB antibody (Proteintech 18195-1-AP).
Previous structure-focused studies have shown that LACTB forms filaments of varying lengths and undergoes multiple maturation steps resulting in several LACTB bands visualized by Western blot. Additionally, during the preparation of recombinant enzymes like LACTB, serine protease inhibitors cannot be used, as they may also inhibit the isolated enzyme itself, potentially rendering it non-functional. As a result, some degree of protein degradation is commonly observed in recombinant enzyme preparations.

In light of our findings in this study, we observed that LACTB undergoes slight autoproteolysis after being isolated from cells, even when stored at 4°C. This enzymatic activity is notably enhanced when incubated at 25°C, as demonstrated in **Figure 1C**. It is important to note that the antibody used for detection was rigorously validated. The manufacturer, as well as independent users through official reviews, confirmed its specificity. Furthermore, we conducted our own validation through overexpression and downregulation studies. To ensure accuracy, the Western blot bands were excised from the gel and analyzed via mass spectrometry, which confirmed that the detected protein was indeed LACTB. Given this thorough validation process, we are confident that the antibody used is highly specific for LACTB.

The assay shown in **Figure 1C** is a Western blot, which is referenced in the manuscript text (line 404) as follows: "*We incubated recombinant… in PBS buffer and examined the resulting stability of LACTB by Western blot (Fig. 1C)*," and is also mentioned in the Materials & Methods section (line 189). To ensure clarity, we have now added this information to the figure legend as well. The antibody used for the assay was the LACTB rabbit polyclonal antibody (Proteintech 18195-1-AP). A complete list of antibodies, including dilutions, catalog numbers, and other relevant details, can be found in **Supplementary Table 4** appended at the end of the manuscript. In response to the reviewer's suggestion, we thoroughly reviewed the manuscript and have updated all figure legends, text, and the Materials & Methods section with more detailed experimental information.

**2.** Fig 2 – Error bars for panel C would be important to include. Mitochondrial markers as part of the analysis shown need to be included (i.e., mitotracker as shown in Fig 3). The difference in tumor growth presented in panel G is about 20% (panel G). How specific is the AAPA+TVGA for LACTB? Can authors exclude the possibility that this inhibitor affected additional targets?

<u>**Response:**</u> We have added error bars to previous **Figure 2C** (**Fig. 2B** in the revised manuscript). In **Figure 2E** (**Suppl Fig. 3A** in the revised manuscript), we did not use Mitotracker; instead, we employed antibody staining for mitochondrial surface proteins, which serves as an effective mitochondria marker and is commonly used in many studies (Merck, MAB1273). We have now updated the figure legend to include this information.

The difference in tumor growth between tumors expressing wtLACTB and those expressing a mutated version of LACTB, referred to as LACTB-AAPA+TVGA, is approximately 20% during the early stages of LACTB expression. However, as tumor growth progresses, it becomes evident that the LACTB mutant, which lacks autoproteolytic activity (termed LACTB-AAPA+TVGA), demonstrates stronger tumor-suppressive effects than the wtLACTB counterpart. This is shown by the complete disappearance of some tumors, while the remaining tumors are reduced in size by 20-30% compared to tumors expressing wtLACTB. This represents a highly significant effect, with a p-value of $< 0.0001$, denoted by four stars on the graph.

To clarify the specificity of AAPA+TVGA for LACTB and address concerns regarding potential off-target effects, we would like to correct the reviewer: AAPA+TVGA is not an inhibitor. Rather, it is a mutant of LACTB that contains the AAPA and TVGA mutations, replacing the AAPD and TVGD sequences found in the wild-type LACTB. As such, this mutant is specific to LACTB.

**3.** Fig 3 – the focus on MRPS34 is not clear. Authors claim (line 549) that "binding partners identified through hypothesis-driven literature search," yet who were there and how they were selected remains unknown. Data in panels F, G, and H, I also need to include levels for LACTB. Likewise, relative expression levels of the two proteins in patient data need to be shown. Panels H and I are not labeled in the figure.

**Response:** We apologize for the confusion and realize we did not explain ourselves clearly in that sentence. MRPS34 was identified by mass spectrometry as one of the proteins pulled down with LACTB. We performed two biological replicates of this experiment, and MRPS34 was detected in both, making it one of the few common mitochondrial hit proteins (the mass spectrometry data and analysis are included in **Supplementary Data 2**). Among the common mitochondrial hit proteins, we decided to focus further on MRPS34 because a previous study in the literature showed that LACTB is associated with mitochondrial ribosomal complexes, although it did not specify which particular ribosomal protein (reference 41 in our manuscript). We have revised the sentence to read: "*The proteins identified through mass spectrometry (Suppl. Data 2) were then validated for their ability to bind LACTB via secondary immunoprecipitation and subsequent Western Blot analysis. The results showed that mitochondrial small ribosomal subunit protein mS34 (MRPS34) was efficiently….*" (line 484).

Former **Figures 3F-I** (now **Suppl Fig 4C-F** in the revised manuscript) show the RNA expression of MRPS34 across different tumor types, and stages and subclasses of breast cancer, based on bioinformatics analysis of TCGA RNA expression datasets. These results indicate that MRPS34 is highly expressed in cancer tissues, as assessed through RNA-directed experiments. We did not include LACTB levels in this analysis because they are not informative at the RNA level. As previously discussed, and as indicated in this study, LACTB is regulated post-transcriptionally at the protein level, not at the RNA level, and the TCGA database does not provide information regarding protein levels.
In **Figure 3H** (now **Figure 4E** in the revised manuscript), we extended this observation to our panel of breast cancer cell lines, demonstrating that, similar to tissue samples, MRPS34 RNA levels are also elevated in these cell lines. In response to the reviewer's request, we have included the RNA expression levels of LACTB in the revised manuscript, which show no correlation between MRPS34 and LACTB expression at the RNA level (**Supplementary Figure 4I**, text line 510). However, as mentioned earlier, this lack of correlation is readily explained, since LACTB is regulated by MRPS34 at the protein level, not the RNA level.
In the former **Figure 3I**, we presented only protein levels of MRPS34, and we agree with the reviewer's suggestion that the protein levels of LACTB should also be included. As a result, these experiments were repeated, and in the revised manuscript, we now show both the endogenous protein levels of LACTB and MRPS34 in a single Western blot image (**Figure 4F**, included is the quantification of the Western Blot image). The results from this Western blot confirm the inverse relationship between the protein expression levels of LACTB and MRPS34.

We correctly labeled all the panels in the presented figures, thank you for notifying us.

Regarding the relative expression levels of the two proteins in patient data, we obtained human breast clinical tissues (n=119) from normal and tumorigenic (grade 1, 2, and 3) origins. We performed immunohistochemistry on these tissues, staining them with LACTB and MRPS34 antibodies. The results revealed an inverse correlation between the expression levels of LACTB and MRPS34 in cancer tissues (with comparisons made within the same tissue slices). Specifically, in tissues where MRPS34 expression was high, less LACTB expression was observed. This dataset is presented in **Figure 4G** (line 512) of the revised manuscript. These findings support the data obtained from breast cancer cell lines and extend our observations to more physiological human primary tumor models. They also highlight the role of MRPS34 in negatively regulating LACTB, a relationship that is explored in greater detail in the follow-up **Figure 5**.

**4.** Figure 4 – data are shown for LOS and OE (panels A, B), but the actual level of endogenous proteins is not shown and is required. The data presented can not exclude the indirect effect of MRPS34 on LACTB. Likewise, the stoichiometry for these proteins in vivo is important to establish and recapitulate in vitro assays. 30h is needed to identify a notable change in LACTB (panel C), which is concerning and questions the physiological significance of the interaction. The changes shown for the proliferation of breast and

ovarian cancer cell lines upon treatment with MRPS34 are rather small (panel D), raising concerns regarding the physiological significance of these observations.

**Response:** We would like to clarify for the reviewer that the endogenous protein levels are always shown in both figures, as it is essential for drawing conclusions. In **Figure 4A (Figure 5A** in the current manuscript), the lanes labeled "MCF7R," "T47D," "HS578t," "HCC1806," and "HCC38" represent control cells, showing the endogenous levels of the proteins we are immunoblotting for. Adjacent to these control lanes are the manipulated cells, labeled "MCF7R_shMRPS34," "T47D_shMRPS34," "HS578t_shMRPS34," "HCC1806_shMRPS34," and "HCC38_shMRPS34," in which MRPS34 was knocked down.
The same applies to **Figure 4B**: when labeled "no dox," it indicates that the transduced protein is not being overexpressed, and therefore, the expression levels represent the endogenous levels of the studied protein. To prevent any future confusion, we have updated the figure legends to mention the presence of control cells.

The reviewer is correct in noting that this figure does not exclude the possibility of an indirect effect of MRPS34 on LACTB. However, it does demonstrate an important biological effect that cannot be dismissed: the inverse expression pattern of these two proteins. Given that we also questioned whether their interaction is direct or indirect, we included additional experiments throughout the manuscript to address this. For example, dot blot analysis and proximity ligation assays (PLA) were conducted to investigate their direct interaction. In **Figure 4B**, we performed *in vitro* dot blot analysis using purified recombinant LACTB and MRPS34, demonstrating that these two proteins directly bind to each other. In **Figure 4C-D**, we extended these experiments to *in vivo* cellular settings, employing recently developed PLA microscopy. This technique detects only very close interactions between proteins (less than 40 nm) and is widely regarded in the field as strong evidence of direct interaction.
Furthermore, other figures, such as **Figure 5C**, which show the effect of MRPS34 on LACTB autoproteolysis, were performed using recombinant proteins. Recombinant LACTB and MRPS34 were incubated together in these experiments, further supporting the claim for direct interaction between these two proteins.
However, whether the interaction between LACTB and MRPS34 is direct or indirect, our claim that MRPS34 is a negative regulator of LACTB and can enhance LACTB autoproteolysis remains valid in both scenarios.

Regarding the stoichiometry of these proteins *in vivo*, we indeed performed mass spectrometry experiments to determine the ratio of LACTB and MRPS34 in cancer cells prior to conducting our *in vitro* assays. The result of this mass spectrometry experiment was not included in the manuscript. In this experiment, we analyzed the ratio of LACTB and MRPS34 proteins in non-tumorigenic HME cells and tumorigenic MCF7ras cells (**Fig. R1** for reviewer only). Our results showed that in MCF7ras cancer cells, the MRPS34/LACTB ratio was significantly greater than in HME cells, which supports our findings from cancer cell lines showing higher MRPS34 expression and lower LACTB expression in tumorigenic cells.
Initially, in our *in vitro* assays, we added up to 10-15 times more MRPS34 than LACTB to mimic the ratio of these proteins *in vivo*. However, during further optimization, we observed that we obtained the same *in vitro* results even when the ratio of these proteins was 3:1 (MRPS34/LACTB). Consequently, to conserve recombinant protein preparations, we proceeded with a 3:1 (MRPS34/LACTB) ratio in our follow-up experiments.

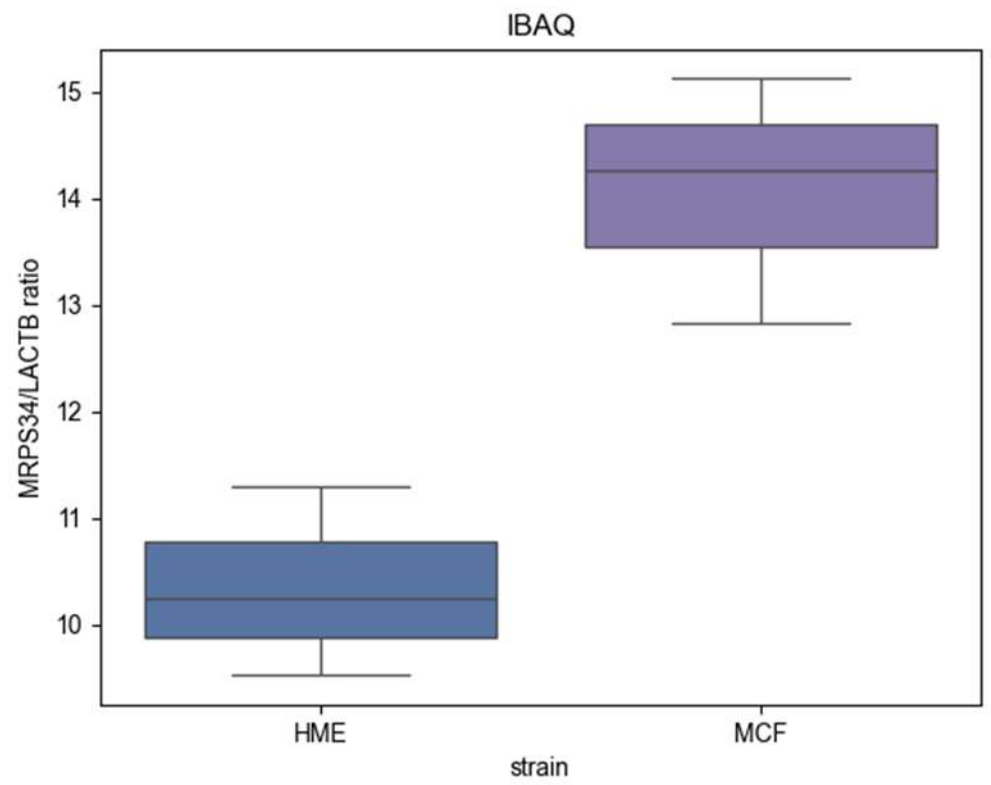


**Figure R1 (for reviewer only)**. Mass spectrometry results examining the stoichiometry of MRP34 and LACTB in non-tumorigenic HME cells and tumorigenic MCF7ras cells.

In **panel 5C**, we indeed see a change in LACTB stability at later time points of interaction with MRPS34, with notable change at 24 hours, and obvious change (almost complete autoproteolytic degradation) at 30 hours. This experiment was performed *in vitro* using purified recombinant proteins (LACTB and MRPS34) incubated together. As such, it is not surprising that under these simplistic conditions the kinetics of the reaction might not represent the kinetics under *in vivo* conditions. Indeed, almost no chemical/enzymatic reaction performed *in vitro* precisely recapitulates the situation inside the cells. The *in vitro* conditions represent surrogate conditions used to find out whether the interaction is possible, whether the two proteins have the ability and potential to influence each other. Our experiments clearly show that these two proteins (LACTB and MRPS34) are indeed influencing each other greatly. We performed these experiments with several other proteins (actin, COQ6, HIGD1A, GAPDH, TOM20) with LACTB and none of them influenced LACTB stability even when incubated together with LACTB for many days (data not shown, but we can include the data in supplementals). The precise kinetics of these reactions is carefully controlled and fine-tuned *in vivo* (in cells) through usage of specific salts, pH, compartmentalization, posttranslational modifications, cofactors and specific binding factors. This amount of complexity can't be reproduced *in vitro* and therefore, while our work shows correctly that these two proteins indeed interact and modulate each other, under in vitro conditions we observe these interactions under slower kinetics. This, however, doesn't change our claim that these two proteins indeed affect each other. The precise kinetics and conditions of this interaction will require several detailed studies which we intend to follow closely in future.
However, it is indeed interesting to observe how the kinetics of LACTB autoproteolytic reaction is increasing under more complex conditions. In our first observations we show (**Fig. 1C**) that when LACTB is incubated on its own (the most simplistic condition) it is capable of autoproteolysis albeit very inefficiently and slowly (at 96 hours of *in vitro* incubation). When we remove some of the salts from the reaction (change PBS to water) this autoproteolytic activity is increased and now we can observe it at 36-48 hours **(Fig. 1D)**. When we add MRPS34 to this reaction (thus getting even closer to *in vivo* conditions), we further increase the reaction of autoproteolytic activity, which is now observable at 24 hours. So, in this case through modulating the *in vitro* conditions we already increased the kinetics of the experiments from 96 hours to 24 hours. These are indeed nice experiments showing how observations made *in vitro*, while qualitatively correct, are quantitatively modulated and correctly adjusted the more precise and complex is the *in vivo* environment they are performed in. We intend to research the detailed conditions of the interaction (salts, pH, cofactors, posttranslational modifications…) of these two proteins in our follow up work.

In **panel 4D**, we focus solely on breast cancer cell lines: MCF7R and HS578t, not ovarian cells. While the differences in proliferation are small at the early stages of the study, they become more pronounced at later time points and are statistically significant ($p<0.0001$, indicated by four stars in the figure). It is important to note that the y-axis is logarithmic (Log10), meaning that the difference between the conditions is fivefold or greater at the later time points, which represents a substantial difference in proliferation rates.

## Reviewer #2:

We thank the reviewer for the insightful comments and for recognizing the potential interest of our findings in the scientific community. We appreciate the opportunity to revise our manuscript and to address the reviewer's concerns through additional experiments, clarifications, and discussion.
The reviewer's comments are highlighted in gray.

**Comments:**

**1.** While the authors claim to identify LACTB among a panel of 15 mitochondrial proteins which are putative substrates of LACTB these other targets and Non-Targets are not further evaluated nor used as positive nor negative controls for their subsequent assays.

**Response:** The other hits were not ignored; we are actively investigating them. However, from the beginning, we focused our attention on LACTB because it was one of the most compelling hits, and the idea that LACTB might be cleaving itself was particularly fascinating. In scientific research, once a list of candidate proteins is identified through rigorous and established criteria such as multiple biological replicates, p-values, and fold-changes, the final selection for in-depth validation is left to the discretion of the researcher. There is no requirement, either scientifically or from the publishing community, to test all identified hits within a single manuscript. In fact, doing so would be impractical in terms of space, funding, and clarity. Our decision to focus on LACTB does not diminish the significance of the discoveries presented in our manuscript. The findings stand on their own, contributing valuable insights to this line of research.

**2.** In Figure 1 the authors describe and execute a modified PICS assay to identify putative substrates of LACTB. They then go on to show via Western blot that recombinant LACTB wt but not an inactive mutant S164A is degraded at 4 days. Unfortunately the Coomassie used in the supplemental figure is under developed so that readers cannot see any bands beyond the dominant monomer, while the Western shows an array of bands including some appearing at the size of the degradation product highlighted in figure 1. This perturbs an otherwise troubled initial claim in as much as all of the other conditions show inhibited LACTB activity while there is no way to gauge the accumulation of the cleavage product over time as the data currently stands in either the main figure nor the supplement and the band highlighted at 4 days is present in every condition though seemingly enriched in the H20 at 36 hrs. The arguments are further destabilized by the perceived lack of specificity of the antibody which shows multiple bands in all the blots even in supplement where they are showing their input protein for the assays where there is only a single band in the coomassie. Perhaps a silver stain or other more sensitive approach would be more appropriate here so that readers can compare the bands observed in the western to the initial proteins present in the tube.

**Response:** The purpose of the Coomassie staining (shown in the left panel of Suppl. Fig. 1A of the previous version of the manuscript) was solely to demonstrate that equivalent amounts of the two isolated proteins (wild-type LACTB and a control catalytic mutant of LACTB, referred to as S164A LACTB) were used in the PICS assay. This figure was not intended to show the autoproteolytic activity of LACTB, as the reviewer correctly pointed out. The Coomassie staining experiment was performed immediately after protein isolation, within a few hours. However, as shown in **Fig. 1C** (and discussed by the reviewer), the autoproteolysis of wild-type LACTB occurs with slow kinetics at much later time points (at 4 days of incubation). To prevent further confusion, we have removed this part of the Supplemental Figure. We address concerns regarding antibody specificity two paragraphs below.

Regarding the next reviewer's comments, the figure demonstrating LACTB's ability to undergo proteolysis—albeit inefficiently at 4 days—was not intended to depict the kinetics of cleavage product appearance over time. Rather, this initial experiment establishes that wild-type LACTB is capable (has the potential) to cleave itself, though inefficiently, as also noted in the text of the manuscript (line 413). The cleavage becomes evident only after 4 days of incubation, making this a qualitative rather than a quantitative figure.

Additionally, the figure includes two negative controls: one where LACTB's enzymatic activity is inhibited pharmacologically (via a chemical inhibitor) and another where it is inhibited genetically (using the catalytic mutant S164A-LACTB). The image clearly shows that, after 4 days, the catalytically active monomeric and polymeric forms of LACTB (wtLACTB) disappear completely (a robust result, further highlighted by the red arrow), while several new degradation bands emerge. Since no changes are observed on days 1 and 2, these time points serve as references, illustrating the protein's state before evident autoproteolysis occurs.

The kinetics of LACTB autoproteolysis is addressed in **Fig. 5C**, which demonstrates its acceleration when incubated with MRPS34 across six different time points.

**Figure 1C** depicts recombinant LACTB protein, which was obtained by pulling down, purifying, and concentrating LACTB expressed in mammalian HEK293T cells. The protein was visualized via Western blot using a polyclonal LACTB antibody (Proteintech 18195-1-AP).

Previous structure-focused studies have shown that LACTB forms filaments of varying lengths and undergoes multiple maturation steps resulting in an array of LACTB bands visualized by Western blot. Additionally, during the preparation of recombinant enzymes like LACTB, serine protease inhibitors cannot be used, as they may also inhibit the isolated enzyme itself, potentially rendering it non-functional. As a result, some degree of protein degradation is commonly observed in recombinant enzyme preparations. Moreover, throughout our study, we observed that LACTB undergoes slight autoproteolysis after being isolated from cells, even when stored at 4°C. This enzymatic activity is notably enhanced when incubated at 25°C, as demonstrated in **Figure 1C**.

It is important to note that the antibody used for detection was rigorously validated. The manufacturer, as well as independent users through official reviews, confirmed its specificity. Furthermore, we conducted our own validation through overexpression and downregulation studies. To ensure accuracy, the Western blot bands were excised from the gel and analyzed via mass spectrometry, which confirmed that the detected protein was indeed LACTB. Given this thorough validation process, we are confident that the antibody used is highly specific for LACTB.

**3.** In Figure 2 the authors set out to identify the cleavage sites in LACTB identifying the AATD and TVGD peptides as the most efficiently cleaved by LACTB. While the authors argue that the variants retain their full enzymatic activity (S2D) they do not compare this activity against wt only the S164A variant making comparisions difficult a feature that is further exaserbated by the difference in WT activity shown at 1 and 2 days in this figure as compared to that shown in figure 1. Futhermore there is no data in the figure nor supplement that shows that the WT and AATD/TVGD constructs are expressed or purified at the same levels limiting the utility of the authors comparisons given that they are overexpressing a known tumor suppressor. DESPITE the authors claims to the contrary. This makes the interpretation of 2E-H non-feasible. Are the tumors that never grew (even before initiation of the perturbation being counted in the quantification/statistics)? If so how is that justified here?

**Response:** We acknowledge the reviewers' concerns and have added the missing data. **Suppl. Fig. 2C** of the revised manuscript demonstrates that the AAPA+TVGA mutant of LACTB retains its catalytic activity against an external peptide very similar to that of wtLACTB. **Fig. 2D** more clearly and informatively

illustrates the inability of the AAPA+TVGA LACTB mutant to undergo autoproteolysis when compared to wtLACTB. Notably, these images also demonstrate the complete absence of small molecular forms of LACTB (below the LACTB monomer). This confirms that these lower bands indeed represent LACTB peptides rather than nonspecific bands. These peptides might represent shorter physiological forms of LACTB, which may arise from proteolytic processing and maturation of the LACTB enzyme or LACTB filaments or through ongoing/residual LACTB autoproteolysis occurring during recombinant wtLACTB preparation. The identity of these bands as LACTB-derived peptides was confirmed early in our studies through mass spectrometry analysis.

Regarding the next concern we would like to correct the reviewer that we indeed showed (in **Suppl. Fig. 3B**) the expression levels of both LACTB-WT and LACTB-AAPA+TVGA in both cell types that we used for the *in vivo* experiments (MCF7ras, HCC1806). In fact, LACTB-AAPA+TVGA in MCF7ras was a little less expressed than LACTB-WT, and yet it showed a stronger effect in halting cell proliferation and *in vivo* tumor growth. To avoid further confusion, we emphasized this data in text (line 458).

In our analysis, we included all tumors. Even though two tumors in the LACTB-AAPA+TVGA group were very small (0.02 cm) before perturbation, they were still counted. We were careful not to exclude any data points from the analysis or statistics due to ethical considerations. Any tumor that formed, regardless of size, was included. In past experiments, we have observed that even initially small and slow-growing tumors can later accelerate in growth.
Additionally, it is possible that our doxycycline-inducible system exhibits slight leakage under *in vivo* conditions, leading to low-level expression of wtLACTB and LACTB-AAPA+TVGA even in the absence of doxycycline. However, the fact that even such small amounts of leakage would result in smaller initial tumors in LACTB-AAPA+TVGA group than in wtLACTB group further supports the conclusion that LACTB-AAPA+TVGA has a more pronounced inhibitory effect on tumor growth compared to its wild-type counterpart.

**4.** Figure 3: The authors argue that LACTB binds to MRPS34. 2A shows a faint band in the CONTROL IP for MRPS34 which is reciprocally present when they do the inverse pulldown with MRPS34 making it difficult to interpret. 2B lacks input and IgG controls for each condition tested. It would greatly strengthen the argument if the authors provided LACTB levels for all of the cell lines tested in the (unlabeled) portion of this figure.

**Response:** Yes, we observed a faint band in the control, which may be due to inadequate washing or nonspecific binding of MRPS34 to the beads. However, we clearly demonstrate that the MRPS34 band is significantly stronger (at least sevenfold) when pulled down with the LACTB antibody, leading us to conclude that MRPS34 and LACTB may form a complex.
To further validate this observation, we conducted two additional experiments 1. **Fig. 4B**: We confirmed the interaction between MRPS34 and LACTB using dot blot analysis. This assay, performed with purified recombinant LACTB and purified recombinant MRPS34, demonstrated that the two proteins directly bind to each other. 2. **Fig. 4C-D**: We extended this finding to cellular experiments by employing proximity ligation microscopy, a technique that detects only very close protein interactions (less than 40 nm). In the field, this method is considered strong evidence of direct interaction.
Based on these three independent observations, performed both *in vitro* and in cells using well-established molecular biology techniques, we concluded that MRPS34 and LACTB can be a part of the same complex.

We agree with the reviewer that including LACTB protein levels in this Figure is important. Therefore, we repeated these experiments, and in the revised manuscript, we now present both endogenous LACTB and MRPS34 protein levels in a single Western blot image (**Figure 4F**, included is the quantification of the

Western Blot image). The results from this Western blot confirm the inverse relationship between the protein expression levels of LACTB and MRPS34.

**5.** Figure 4: In A the Blots are uneven and difficult to interpret in order to confirm the authors claims. Quantification (error bars) is inappropriate given the reported n of 2. These issues are compounded/ even worse in B and the corresponding supplement which do not seem to align with the authors claims about the relationship between MRPS34 and LACTB protein levels.

**Response:** Figure 4 is Figure 5 in the current revised manuscript. The Western blot and quantifications have been corrected, and for certain parts, we prepared new samples and new Western blots, which we believe are now clearer and easier to interpret. In blot A, the red arrows highlight the increase in endogenous LACTB levels across several different cancer cell lines following the downregulation of endogenous MRPS34 protein. Quantification of both **Fig.5A** and **5B** was corrected.
Notably, since MRPS34 is part of the mitochondrial ribosomal complex, complete knockout is not feasible (as we and others have observed). Therefore, we worked with partially decreased MRPS34 levels. Despite only a partial reduction in MRPS34, we observed a clear increase in endogenous LACTB levels across five tested cell lines, demonstrating the generality of this inverse relationship across various types of breast cancer. These new experiments further confirm the inverse expression pattern of LACTB and MRPS34 in a wide range of cancer cell lines.

## Reviewer #3:

We thank the reviewer for the informative comments and for acknowledging that our study contains significant advancements. We are grateful for the opportunity to revise our manuscript and to address the reviewers' concerns through additional experiments, clarifications, and discussion.
Reviewer's comments are highlighted in grey.

**Major points:**

**a.** The authors do not adequately demonstrate that LACTB undergoes auto-proteolysis in cells or cancer cells (see below point 5)

**Response:** Addressed below in point 5.

**b.** The link with MRSP34 needs to be strengthened using appropriate controls (see below).

**Response:** Addressed in point 10 and the follow-up points.

**c.** It is essential to show that the catalytic inactive LACTB mutant (C124S) is not able to induce autoproteolysis in Figure 1C-D or Supplement 1 (and later figures). This will confirm that it is LACTB-dependent proteolysis and not a contaminant. Consider using LACTB KO cells to ensure that there is no dimerization with endogenous LACTB in these experiments.

**Response:** A small correction for the reviewer: the catalytically inactive mutant is called S164A, not C124S. We also consider this experiment essential for the same reasons as the reviewer. In fact, we included this important control—the catalytically inactive S164A LACTB mutant—in **Fig. 1C** at both time points. Our results show that while wild-type (wt) LACTB monomers and multimers are completely degraded after four days of incubation, the S164A catalytically inactive LACTB monomer remains unchanged and intact. This finding rules out the possibility of a protease contaminant in our experimental conditions, as all proteins were isolated and tested under identical conditions using the same buffers on multiple occasions.

If contaminating proteases were present in our preparation, buffers, or experimental setup, we would expect to see full degradation of the S164A LACTB mutant as well (since it differs from wtLACTB only in one amino acid change). These experiments early on in the manuscript demonstrate the necessity of intact catalytic activity for LACTB autoproteolysis, therefore our follow-up studies focused on working with wtLACTB—both *in vitro* (using recombinant wtLACTB protein) and *in vivo* (using endogenous, overexpressed, or downregulated wtLACTB).
Regarding LACTB knockout (KO) cells, unfortunately, generating them is not feasible. It has been shown by us and others that severe downregulation or knockout of LACTB is detrimental to the viability of both tumorigenic and non-tumorigenic cells. As a result, throughout our work, we have only been able to work with partial knockdowns using shRNA. One proposed role of LACTB is structural, maintaining mitochondrial membrane and cristae organization through filament formation. Severe downregulation or knockout of LACTB could therefore lead to mitochondrial collapse. While this poses technical challenges for LACTB research, preventing the clear, black-and-white experimental results that full knockouts might provide, it has important implications for cancer therapeutics. Since cancer cells cannot completely eliminate LACTB, they must actively suppress its expression to counteract its tumor-suppressive function therefore allowing us to find feasible ways to revert this process. This is further supported by the absence of loss of heterozygosity (LOH) or detrimental LACTB mutations in cancer cells. Consequently, LACTB is considered an example of a haploinsufficient tumor suppressor, with strong therapeutic potential for restoring its protein levels in cancer cells - one of the key goals of our future work.
While we were unable to fully knock out LACTB, we acknowledge the possibility that during the preparation of the recombinant S164A LACTB mutant, some wtLACTB may have been co-purified due to dimerization. However, the mutant protein was overexpressed at much higher levels than endogenous wtLACTB in HEK293T cells, which were used for recombinant protein isolation. As a result, any residual "contaminating" wtLACTB would be present at minimal levels and unlikely to interfere with the main conclusion of **Fig. 1C**, which shows that the S164A mutant remains intact at tested time points.

**d.** The text needs to be carefully in many places to avoid describing LACTB's autoproteolytic ability – which is presumably a reflection of its catalytic activity. Suggest instead using the description non-cleavable LACTB and be careful to differentiate between catalytic inactive and non-cleavable LACTB. For example, in the abstract line 41 and 42 – it sounds as though the "autoproteolytic activity" of LACTB is required for tumour suppression activity but, according to the author's model, it is the opposite – it negatively affects its tumour suppressor activity.

**Response:** We thank the reviewer for this suggestion. Indeed, the autoproteolytic activity of LACTB is a subset of its enzymatic activity. We had struggled to find precise terminology to distinguish the three different catalytic states of LACTB, and our previous descriptions were somewhat cumbersome. We have now implemented the reviewer's suggestion throughout the manuscript, using the terms **wild-type LACTB, catalytically inactive LACTB, and non-cleavable LACTB** for clarity.
Additionally, we agree that the statement in the abstract (line 41), *"that LACTB possesses autoproteolytic ability, which is important for its tumor suppressor activity,"* was somewhat misleading due to a wording issue. To clarify our intended meaning, we have revised it to: *"that LACTB possesses autoproteolytic ability, which is important for modulating its tumor suppressor activity."*(line 29).

**Other comments/Minor points:**

**1.** Figure 1B: Quantitative information about the mass spectrometry analysis is required, including the n values and quantitative/statistical values. This also applies to Figure 3 – for which no information is provided about the mass spectrometry experiment used to identify MRSP34. Including the dataset will allow the reader to understand if MRSP34 is a potential LACTB substrate for example is MRSP34 in Figure 1B and in Figure 3A?

**Response:** The reviewer is correct, we did not include the mass spectrometry files in the initial submission, only the final tables and results. We have now added this information to the supplemental material (**Suppl. Data 1** and **Suppl. Data 2**).
In summary, MRPS34 was identified as a binding factor in our immunoprecipitation experiments through mass spectrometry. However, it was not found to be a substrate of LACTB in our PICS assay followed by mass spectrometry analysis. To ensure we did not overlook anything, we incubated these two proteins together under various *in vitro* conditions. We never observed MRPS34 being degraded by LACTB. Instead, our results showed the opposite, MRPS34 interacted with LACTB and enhanced LACTB autoproteolysis, as described in this manuscript.

**2.** Figure 1C-D: It would be easier to compare protein levels if referenced to time point 0. For example, is there significant proteolysis over 24 hours? Additionally, including all samples on a single gel would facilitate the comparison of absolute protein amounts. Also, what does the red arrow on the gel indicate?

**Response:** In this figure, we included a limited number of time points. For **Fig. 1C**, we selected two time points (2- and 4- days) in PBS, while **Fig. 1D** includes a single time point (36 hours) in PBS and $H_2O$. These initial qualitative figures were designed to demonstrate LACTB's ability to undergo autoproteolysis.
A more detailed quantitative time-course study is presented in **Fig. 5C**, which serves as a fine-tuned follow-up to **Fig. 1D**. In **Fig. 5C**, we examined LACTB autoproteolysis at 6-hour intervals (18, 24, 30, 36, 42, and 48 hours) in $H_2O$, allowing direct comparison with **Fig. 1D** rather than **Fig. 1C**. The first significant autoproteolytic activity was observed at 24 hours when LACTB was incubated with MRPS34 (**Fig. 5C**). At the 18-hour time point, no significant autoproteolysis was detected.
The earlier time points across these figures serve as important controls, demonstrating equal protein loading and showing the initial state of LACTB before robust autoproteolysis begins. These time points effectively act as negative controls, comparable to time point 0, before the protein is incubated at 25°C.
We did not include all samples on a single gel because, in our experience, multi-well gels (above 12 wells) do not provide sufficient band separation—closely migrating bands tend to smear, and only small protein volumes can be loaded. However, the key comparisons in **Fig. 1C** and **1D** were qualitative within individual time points, rather than quantitative comparisons across different time points. The small increase in total protein levels on Day 4 compared to 2 is likely due to variations in membrane transfer efficiency or exposure differences, but this does not impact the key conclusions drawn from within each time point. The red arrow in the figure highlights the complete disappearance of the LACTB monomer, and we have now included this clarification in the figure legend.

**3.** Figure 1: As above, it is essential to show that the catalytic inactive LACTB mutant (C124S) is not able to induce autoproteolysis in Figure 1C-D or Supplement 1 (and later figures).

**Response:** Answered in point **c** above.

**4.** Figure 2D: The expression of LACTB-wt and AAPA-TVGA is wildly different. It would be important to use inhibitor Z-AAD-CMK with AAPA-TVGA (as in figure 1C) to prove that AAPA-TVGA is not cleaved. As above, when protein samples are not directly compared on the same gel, it is challenging to assess whether the AAPA-TVGA variant remains unchanged or decreases compared to day 1.

**Response:** We agree with the reviewer and have repeated these experiments, incorporating all suggested changes, ensuring comparable expression, and running the samples on the same gel. The updated results are now included in **Fig. 2D**. These results confirm that the AAPA+TVGA LACTB mutant is unable to

undergo autoproteolysis, further strengthening our original claim in a more informative way. Notably, these images also demonstrate the complete absence of small molecular forms of LACTB (below the LACTB monomer). This confirms that these lower bands indeed represent LACTB peptides rather than nonspecific bands. These peptides might represent shorter physiological forms of LACTB, which may arise from proteolytic processing and maturation of the LACTB enzyme or LACTB filaments or through ongoing/residual LACTB autoproteolysis occurring during recombinant wtLACTB preparation. The identity of these bands as LACTB-derived peptides was confirmed early in our studies through mass spectrometry analysis.

**5.** Figure 2: To appropriately reach the author's model and to interpret the proliferation rates in F and the tumour size in G, additional experiments and detailed data are required. Some of this information is briefly shown in the supplementary figures but needs to be presented in greater detail. Specifically:

a)b) There should be an effort to show that WT-LACTB is cleaved in human cells, not just in vitro. This can be done by showing cleavage products running at appropriate sizes using N- and C-terminal tags and antibodies against different domains of LACTB. Using MG132 can help prevent the degradation of cleavage products. The stability of LACTB-WT and LACTB-AAPA-TVGA in different cell lines should be included encompassing a cycloheximide chase. This would enable the conclusion that the LACTB-AAPA-TVGA levels contribute to the tumour suppression ability. The results as presented are confusing since the ultimate levels of LACTB-AAPA-TVGA (in Supplement 2E) appear to be less than wild-type, which seems to contradict the model.

**Response:** We agree with the reviewer, we followed the advice and in the revised manuscript we provide evidence that WT-LACTB is also cleaved in a cellular context, in human cells. We expressed doxycycline inducible LACTB-WT and LACTB-AAPA+TVGA for one day in MCF7ras and HCC1806 cells and examined by Western blot the appearance of the degradation products of LACTB. Indeed, expression of LACTB-WT led to appearance of several degradation products while this was not observed in case of the expression of the non-cleavable LACTB-AAPA+TVGA mutant (**Fig. 2E**). The faint degradation bands present in the LACTB-AAPA+TVGA mutant samples are the result of the endogenous LACTB-WT that is present in the small amounts in both MCF7ras and HCC1806 cells as shown in **Fig. 4F**. Moreover, upon treatment of these cells with MG132 proteasome inhibitor we observed increased accumulation of the LACTB-WT degradation products in both cell lines (**Fig. 1E**). These results, which were shown in two cancer models, confirmed that the autoproteolytic activity of LACTB is indeed needed for the generation of LACTB-derived degradation products, that the autoproteolytic activity is physiological and used in cellular setting (is not an artefact of our *in vitro* culture) and that the antibody we are using is specific for LACTB. We also attempted to use antibodies against different domains as suggested by the reviewer. There are limited sources of antibodies against LACTB and almost all of them are polyclonal antibodies against big portion of LACTB (almost the whole protein). However, the few companies that make monoclonal antibodies refused to share the sequence of the antigen peptide with us claiming proprietary information therefore we were not able to use antibodies against different domains of LACTB.
Regarding the supplement 2E (now **Suppl. Fig. 2B** in the revised manuscript). This image shows levels of forced doxycycline-induced exogenous LACTB-WT and LACTB-AAPA-TVGA mutant not the levels of the endogenous proteins (there is no LACTB-AAPA-TVGA mutant endogenously expressed in cancer cells). We carefully titrated the viral vectors and doxycycline levels when introducing these exogenous proteins into cells to ensure comparable levels of their expression (or even slightly lower expression of the mutant) to show that when these levels are comparable (or when the mutant levels are even lower than those of wild type LACTB) the mutant still exhibits stronger tumor suppression (as shown in **Fig.3** and **Suppl. Fig. 3**) than wtLACTB.

c) Ultimately, LACTB-AAPA-TVGA(+C124S) --- incorporation of non-cleavable and also catalytic dead (C164A) --- is needed to understand whether LACTB activity is needed and whether other substrates are important for this phenotype.

**Response:** As per the reviewer's request, we generated the LACTB-AAPA-TVGA(+S164A) mutant, but we were unable to express it in cells. We tested multiple non-tumorigenic and tumorigenic cell lines, re-cloned the mutant into different vector backbones (both constitutive and doxycycline-inducible expression systems), yet the mutant was never expressed. We are unsure of the exact reason for this, but it appears that cells do not tolerate this mutant well. It is possible that these mutations cause such profound structural changes in LACTB that the protein is immediately misfolded and degraded by cellular machinery. However, all our previous data—both in this manuscript and prior studies—demonstrate that the catalytically inactive S164A LACTB mutant cannot cleave any tested peptides that wild-type LACTB cleaves, lacks autoproteolytic activity, and fails to suppress tumor growth. Given this, we are uncertain what additional mutations (such as AAPA+TVGA) would achieve in this background. However, we do not believe they would restore any of the functions that are absent in the S164A LACTB mutant that lacks the catalytic residues.

**6.** Supplement 2D – compare with WT?

**Response:** Done. Please see updated **Suppl Fig. 2C**. It supports our previous claim that AAPA+TVGA LACTB mutant retains catalytic activity similar to that of wtLACTB.

**7.** Supplement 2E – see above, point 5b)

**Response:** Explained in point 5b.

**8.** Figure 3. No validation of the Alphafold2 model is shown. Based on Alphafold2 model, the authors should generate MRPS34 that does not bind to LACTB (and vice versa) to prove their hypothesis and mechanisms. They can use these controls in figure 4B and importantly in figure 4C. I am concerned about contaminating proteases in the in vitro assays.

**Response:**
In our study we performed protein docking to predict the interaction between LACTB and MRPS34. The predicted models show binding of MRPS34 to LACTB tetramer and the formation of a salt bridge between Asp186(LACTB) and Arg101(MRPS34) and numerous other strong interaction sites formed by hydrophobic interactions (**Suppl. Fig. 4B**). To answer reviewer's question, we generated a mutant of LACTB where one of the predicted interacting residues (Asp186) was mutated to alanine. Mutating all the predicted sites at once might generate an unviable mutant. We are now in the process of testing the interaction of this mutant with MRPS34. However, in point 13 the reviewer suggested an alternative experiment to answer this question in the absence of the interaction mutant, which we have followed and provided the resulting data.
Regarding the concern of the reviewer about the contaminating proteases in the *in vitro* assay, we also shared this concern at the initiation of our work. This worry was overruled by our inclusion of the catalytically inactive S164A LACTB mutant in **Fig. 1C**. As explained in more detail above, in point c, the fact that the S164A mutant showed no degradation at any of the time points tested (up to 4 days) showed that there were no contaminating proteases in our experimental conditions being pulled down during the preparation of LACTB recombinant protein. Once we observed the acceleration of the autoproteolytic activity of wtLACTB upon co-incubation with commercially acquired MRPS34 (**Fig. 5C**) we were also wondering whether the commercial preparation technique did not accidentally introduce a contaminating protease into our experimental condition through MRPS34 addition. Therefore, we co-incubated MRPS34

with catalytically inactive LACTB mutant for 48 hours and observed no effect on mutant stability (explained also in point 13, **Fig. R2**). This rules out the presence of contaminating protease also in the MRPS34 recombinant preparation.

**9.** Figure 3A: The IPs are performed with overexpressed LACTB in A but unclear if this is the case in B.

**Response:** Yes, this was also the case in B. However, we removed **Fig. 3B** from the manuscript due to its redundancy.

**10.** Why have the authors picked MRSP34 for their study out of other proteins in the interactome?

**Response:** We apologize for not explaining ourselves clearly in the text. MRPS34 was identified by mass spectrometry as one of the proteins pulled down with LACTB. We performed two biological replicates of this experiment, and MRPS34 was detected in both, making it one of the few common mitochondrial hit proteins (the mass spectrometry data and analysis are included in **Suppl. Data 2**). Among the common mitochondrial hit proteins, we decided to focus further on MRPS34 because a previous study in the literature showed that LACTB is associated with mitochondrial ribosomal complexes, although it did not specify which particular ribosomal protein (reference 41 in our manuscript). We also tested other mitochondrial ribosomal proteins from the list (such as MRPS22 and MRPS29) but we did not observe any direct interaction with LACTB in the follow up experiments as noted in the text (line 489).

**11 and 12.** Statistical significance on the quantification of the blots (Fig.4A-B). The relative upregulation of LACTB is modest. As above, suggest showing the full gel to establish if the cleavage products are reduced. Figure 4B: The gel is not of sufficient quality to assess the result for T47D, For HCC38, the levels of MRSP34 appear to drop at 6 days, when LACTB levels are highest - why is the correlation broken. Quantitate the relative overexpression of MRSP34.

**Response:** The Western blot and quantifications have been corrected, and for certain parts, we prepared new samples and new Western blots, which we believe are now clearer and easier to interpret. In blot A, the red arrows highlight the increase in endogenous LACTB levels across several different cancer cell lines following the downregulation of endogenous MRPS34 protein. The upregulation of LACTB upon MRPS34 downregulation is modest but consistent and was shown in five different cancer cells lines. Notably, since MRPS34 is part of the mitochondrial ribosomal complex, complete knockout is not feasible (as we and others have observed). Therefore, we worked with partially decreased MRPS34 levels. Despite only a partial reduction in MRPS34, we observed a clear increase in endogenous LACTB levels across multiple tested cell lines, demonstrating the generality of this inverse relationship across various types of breast cancer. These new experiments further confirm the inverse expression pattern of LACTB and MRPS34 in a wide range of cancer cell lines (**Fig. 5A-B**)

Regarding the cleavage products, performing this experiment under the cellular experimental conditions that were used in this experiment would not show any degradation products since these would be cleared by the cell. However, per suggestion of the reviewer we performed experiments with the MG132 proteasome inhibitor as explained in point 5a and the results of these experiments performed in two breast cancer cell lines are included in **Fig. 1E**.

Regarding the dropping of the levels of MRPS34 at day 6 (in HCC38 cells) and its effect on LACTB in **Fig. 5B**: Even though the MRPS34 levels in our over-expression system dropped at 6 days they are still expressed several fold higher than in the control (no dox) cells what might be sufficient for efficient downregulation of the endogenous levels of LACTB. Moreover, 6 days of forced overexpression represents rather long time point that might lead to adaptation and selection of subpopulations of cells.

**13.** Figure 4C. In the absence of using MRSP34 that does not bind to LACTB as a control, I suggest using the C164S control.

**Response:** We performed this control early on in our studies, but it was not included in the manuscript. In this experiment we used the catalytic mutant of LACTB (AIAK-LACTB) and incubated it in the presence of MRPS34 for several time points. The results showed that the stability of the catalytically inactive mutant is not affected by the presence of MRPS34. It also confirmed that our commercially obtained MRPS34 recombinant protein does not contain any contaminating proteases (**Fig. R2**).

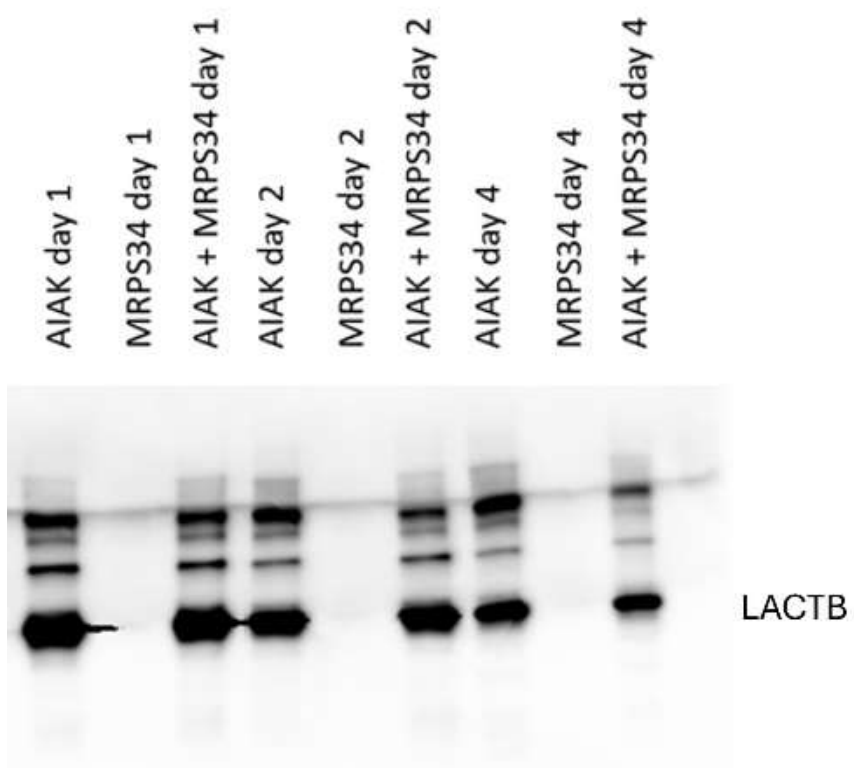


**Fig. R2 (for reviewer only):** Incubation of the catalytic mutant of LACTB (AIAK, where both serines in the catalytic SISK motif were replaced by alanines) with MRPS34 at 25 degrees Celsius for up to 4 days. We occasionally used the catalytic AIAK LACTB mutant as an alternative to S164A mutant (where only the first serine of the SISK residues is replaced by alanine), since we had ample amounts of this mutant at our disposal through our other projects.

Manuscript number: EMBOR-2026-63596V2
Title: The role of autoproteolysis and mitoribosomal proteins in the regulation of LACTB tumor suppressor
Author(s): Zuzana Keckesova, Sara Escudeiro Lopes, Juan Gonzalez-Morena, Alzbeta Baudysova, Michal Svoboda, Pavel Marášek, Gabriel Rodriguez-Gomez, Jessica Ferreira Mendes, Chung Weng Phang, Sravan Kumar Miryala, Pavel Jakoube, Beata Kovarova, Susana Machado, and Yuxiong Feng

Dear Dr. Keckesova

Thank you for your patience while we have reviewed your revised manuscript. We have now received the enclosed reports from referees 1 and 3 of the original manuscript.
The referee both applaud your revision and in principle recommend publication. However, referee 1 rightly points out that since the human data were removed, the manuscript now explicitly over-claims human relevance, for example in the abstract which states 'This study, through in vitro, in vivo, human clinical tumor samples...'. This has to be carefully corrected throughout the manuscript as a condition of publication.
We would also ask you to consider inclusion of the Mass Spec data to address the stoichometry question of ref 1 (#4), which is currently only embedded in your response to the referee comments.

The revised manuscript will undergo a routine quality control and data integrity screen and any issues that arise may be raised subsequently.

Once you have made these minor revisions, please use the following link to submit your corrected manuscript:

Link Not Available

If all remaining corrections have been attended to, you will then receive an official decision letter from the journal accepting your manuscript for publication in the next available issue of EMBO reports. This letter will also include details of the further steps you need to take for the prompt inclusion of your manuscript in our next available issue.

Thank you for your contribution to EMBO reports.

Yours sincerely,

Bernd Pulverer

~~~~~~~~~~~~~~~~~~~~~~~~~~~~~~~~
Bernd Pulverer, Ph.D.
Chief Editor, EMBO Reports
EMBO
Meyerhofstrasse 1, D-69117 Heidelberg
Tel: +4962218891501
bernd.pulverer@embo.org
~~~~~~~~~~~~~~~~~~~~~~~~~~~~~~~~

Referee #1:

The revised manuscript addresses most reviewers comments and is notably stronger. the omission of human data is a minus which detracts from the work presented as claims for relevance are limited. claims for presence of human clinical samples are in the revised manuscript and should be omitted (i.e. abstract).

Referee #2:

This is a comprehensive and well-executed study that proposes compelling mechanisms regulating tumour suppression by LACTB. The authors have made a thorough and good faith effort to address the suggestions of previous reviewers, and I support the publication of this work in EMBO Reports in its current form.

The authors have revised the manuscript as requested and re-submitted.

## 2nd Revision - Editorial Decision 6th Aug 2026

Dear Dr. Keckesova

Thank you for the submission of your revised manuscript to our offices.
I am sorry to undertake yet another round of revision, but there are a couple of remaining issues even after we included a number of minor changes on your behalf.
Since these changes go technically beyond the scope of proofs changes, please incorporate them via a minor additional revision:

- Please note that the exact p values are not provided in the legends of figures 3A, B; 5D, EV3 C, EV4 C, EV5 D. This is stated policy for EMBO publications.
- Please note that information related to n is missing in the legends of figures 4D, E; 5B, EV1 C, EV3 C, EV4 C, D, E, F, I, J.
- Please note that n=2 in figures EV5A & EV4J. it is our policy to exclude stats based on distributions for duplicate experiments as this is meaningless Thus, please show all individual data points along with the mean.
- Please note that the error bars are not defined in the legends of figure EV1 C
- Please note that scale bar is missing for figure EV5 C

Regarding the two additional questions you raised two month ago:
1) thank you for clarifying that 119 biopsies were included. The current abstract is perfectly reasonable. I actually recommend to remove the words 'important new' from the last sentence though as it makes the abstract read like a cover letter.
2) Thank you for including the data in EV3 and 5.

best wishes,

Bernd Pulverer

~~~~~~~~~~~~~~~~~~~~~~~~~~~~~~~~
Bernd Pulverer, Ph.D.
Chief Editor, EMBO Reports
EMBO
Meyerhofstrasse 1, D-69117 Heidelberg
Tel: +4962218891501
bernd.pulverer@embo.org
~~~~~~~~~~~~~~~~~~~~~~~~~~~~~~~~

All editorial and formatting issues were resolved by the authors.

Dr. Zuzana Keckesova
Institute of Organic Chemistry and Biochemistry
Biochemistry
Flemingovo Namesti 2
Prague 12000
Czech Republic

Dear Dr. Keckesova,

I am very pleased to accept your manuscript for publication online and in the next available issue of EMBO reports. Thank you for your contribution to our journal.

Your manuscript will be processed for publication by EMBO Press. It will be copy edited and you will receive page proofs prior to publication. Please note that you will be contacted by Springer Nature Author Services to complete licensing and payment information.

You may qualify for financial assistance for your publication charges. Check your eligibility:
https://link.springer.com/journal/44319/how-to-publish-with-us

Should you be planning a Press Release on your article, please notify the production team via this form in order to coordinate publication and release dates: https://forms.cloud.microsoft/e/eH4A8JVP6g

If you have any questions, please do not hesitate to contact the Editorial Office. Thank you for your contribution to EMBO Reports.

Yours sincerely,

Bernd Pulverer

~~~~~~~~~~~~~~~~~~~~~~~~~~~~~~~~
Bernd Pulverer, Ph.D.
Chief Editor, EMBO Reports
EMBO
Meyerhofstrasse 1, D-69117 Heidelberg
Tel: +4962218891501
bernd.pulverer@embo.org
~~~~~~~~~~~~~~~~~~~~~~~~~~~~~~~~

>>> Please note that it is EMBO Reports policy for the transcript of the editorial process (containing referee reports and your response letter) to be published as an online supplement to each paper. If you do NOT want this, you will need to inform the Editorial Office via email immediately. More information is available here: https://link.springer.com/partners/embo-press/editorial-policies#Peer%20review

## EMBO Press Author Checklist

| | |
|---|---|
| Corresponding Author Name: Zuzana Keckesova | |
| Journal Submitted to: EMBO Reports | |
| Manuscript Number: EMBOR-2026-63596V3 | |

**USEFUL LINKS FOR COMPLETING THIS FORM**
The EMBO Journal - Author Guidelines
EMBO Reports - Author Guidelines
Molecular Systems Biology - Author Guidelines
EMBO Molecular Medicine - Author Guidelines

**Reporting Checklist for Life Science Articles (updated January**
This checklist is adapted from Materials Design Analysis Reporting (MDAR) Checklist for Authors. MDAR establishes a minimum set of requirements in transparent reporting in the life sciences (see Statement of Task: 10.31222/osf.io/9sm4x). Please follow the journal's guidelines in preparing your
**Please note that a copy of this checklist will be published alongside your article.**

**Abridged guidelines for figures**
**1. Data**
The data shown in figures should satisfy the following conditions:
- the data were obtained and processed according to the field's best practice and are presented to reflect the results of the experiments in an accurate and unbiased manner.
- ideally, figure panels should include only measurements that are directly comparable to each other and obtained with the same assay.
- plots include clearly labeled error bars for independent experiments and sample sizes. Unless justified, error bars should not be shown for technical
- if n<5, the individual data points from each experiment should be plotted. Any statistical test employed should be justified.
- Source Data should be included to report the data underlying figures according to the guidelines set out in the authorship guidelines on Data

**2. Captions**
Each figure caption should contain the following information, for each panel where they are relevant:
- a specification of the experimental system investigated (eg cell line, species name).
- the assay(s) and method(s) used to carry out the reported observations and measurements.
- an explicit mention of the biological and chemical entity(ies) that are being measured.
- an explicit mention of the biological and chemical entity(ies) that are altered/varied/perturbed in a controlled manner.
- the exact sample size (n) for each experimental group/condition, given as a number, not a range;
- a description of the sample collection allowing the reader to understand whether the samples represent technical or biological replicates (including how many animals, litters, cultures, etc.).
- a statement of how many times the experiment shown was independently replicated in the laboratory.
- definitions of statistical methods and measures:
  - common tests, such as t-test (please specify whether paired vs. unpaired), simple χ2 tests, Wilcoxon and Mann-Whitney tests, can be unambiguously identified by name only, but more complex techniques should be described in the methods section;
  - are tests one-sided or two-sided?
  - are there adjustments for multiple comparisons?
  - exact statistical test results, e.g., P values = x but not P values < x;
  - definition of 'center values' as median or average;
  - definition of error bars as s.d. or s.e.m.

**Please complete ALL of the questions below.**
**Select "Not Applicable" only when the requested information is not relevant for your study.**

**Materials**

| Newly Created Materials | Information included in the manuscript? | In which section is the information available? (Reagents and Tools Table, Materials and Methods, Figures, Data Availability Section) |
|---|---|---|
| New materials and reagents need to be available; do any restrictions apply? | Not Applicable | |

| Antibodies | Information included in the manuscript? | In which section is the information available? (Reagents and Tools Table, Materials and Methods, Figures, Data Availability Section) |
|---|---|---|
| For **antibodies** provide the following information:<br>- Commercial antibodies: RRID (if possible) or supplier name, catalogue number and or/clone number<br>- Non-commercial: RRID or citation | Yes | Supplementary Table 4 |

| DNA and RNA sequences | Information included in the manuscript? | In which section is the information available? (Reagents and Tools Table, Materials and Methods, Figures, Data Availability Section) |
|---|---|---|
| **Short novel DNA or RNA including primers, probes**: provide the sequences. | Yes | Supplementary Table 2 and 3 |

| Cell materials | Information included in the manuscript? | In which section is the information available? (Reagents and Tools Table, Materials and Methods, Figures, Data Availability Section) |
|---|---|---|
| **Cell lines:** Provide species information, strain. Provide accession number in repository **OR** supplier name, catalog number, clone number, and/**OR** RRID. | Yes | Supplementary Table 1 |
| **Primary cultures:** Provide species, strain, sex of origin, genetic modification status. | Not Applicable | |
| Report if the cell lines were recently **authenticated** (e.g., by STR profiling) and tested for mycoplasma contamination. | Yes | Material and Methods |

| Experimental animals | Information included in the manuscript? | In which section is the information available? (Reagents and Tools Table, Materials and Methods, Figures, Data Availability Section) |
|---|---|---|
| **Laboratory animals or Model organisms:** Provide species, strain, sex, age, genetic modification status. Provide accession number in repository **OR** supplier name, catalog number, clone number, **OR** RRID. | Yes | Material and Methods |
| **Animal observed in or captured from the field:** Provide species, sex, and age where possible. | Not Applicable | |
| Please detail **housing and husbandry conditions**. | Yes | Material and Methods |

| Plants and microbes | Information included in the manuscript? | In which section is the information available? (Reagents and Tools Table, Materials and Methods, Figures, Data Availability Section) |
|---|---|---|
| **Plants:** provide species and strain, ecotype and cultivar where relevant, unique accession number if available, and source (including location for collected wild specimens). | Not Applicable | |
| **Microbes:** provide species and strain, unique accession number if available, and source. | Not Applicable | |

| Human research participants | Information included in the manuscript? | In which section is the information available? (Reagents and Tools Table, Materials and Methods, Figures, Data Availability Section) |
|---|---|---|
| If collected and within the bounds of privacy constraints report on age, sex and gender or ethnicity for all study participants. | Not Applicable | |

| Core facilities | Information included in the manuscript? | In which section is the information available? (Reagents and Tools Table, Materials and Methods, Figures, Data Availability Section) |
|---|---|---|
| If your work benefited from core facilities, was their service mentioned in the acknowledgments section? | Yes | Acknowledgments |

**Design**

| **Study protocol** | **Information included in the manuscript?** | **In which section is the information available?** (Reagents and Tools Table, Materials and Methods, Figures, Data Availability Section) |
|---|---|---|
| If study protocol has been **pre-registered, provide DOI in the manuscript**. For clinical trials, provide the trial registration number **OR** cite DOI. | Not Applicable | |
| Report the **clinical trial registration number** (at ClinicalTrials.gov or equivalent), where applicable. | Not Applicable | |

| **Laboratory protocol** | **Information included in the manuscript?** | **In which section is the information available?** (Reagents and Tools Table, Materials and Methods, Figures, Data Availability Section) |
|---|---|---|
| Provide DOI OR other citation details if **external detailed step-by-step protocols** are available. | Yes | Material and Methods |

| **Experimental study design and statistics** | **Information included in the manuscript?** | **In which section is the information available?** (Reagents and Tools Table, Materials and Methods, Figures, Data Availability Section) |
|---|---|---|
| Include a statement about **sample size** estimate even if no statistical methods were used. | Yes | Material and Methods and Figures |
| Were any steps taken to minimize the effects of subjective bias when allocating animals/samples to treatment (e.g. **randomization procedure**)? If yes, have they been described? | Yes | Material and Methods |
| Include a statement about **blinding** even if no blinding was done. | Yes | Material and Methods |
| Describe **inclusion/exclusion criteria** if samples or animals were excluded from the analysis. Were the criteria pre-established?<br><br>If sample or data points were omitted from analysis, report if this was due to attrition or intentional exclusion and provide justification. | Yes | Material and Methods |
| For every figure, are **statistical tests** justified as appropriate? Do the data meet the assumptions of the tests (e.g., normal distribution)? Describe any methods used to assess it. Is there an estimate of variation within each group of data? **I**s the variance similar between the groups that are being statistically compared? | Yes | Material and Methods and Figures |

| **Sample definition and in-laboratory replication** | **Information included in the manuscript?** | **In which section is the information available?** (Reagents and Tools Table, Materials and Methods, Figures, Data Availability Section) |
|---|---|---|
| In the figure legends: state number of times the experiment was **replicated** in laboratory. | Yes | Figures |
| In the figure legends: define whether data describe **technical or biological replicates**. | Yes | Figures |

## Ethics

| **Ethics** | **Information included in the manuscript?** | **In which section is the information available?** (Reagents and Tools Table, Materials and Methods, Figures, Data Availability Section) |
|---|---|---|
| Studies involving **human participants**: State details of **authority granting ethics approval** (IRB or equivalent committee(s), provide reference number for approval. | Not Applicable | |
| Studies involving **human participants**: Include a statement confirming that **informed consent** was obtained from all subjects and that the experiments conformed to the principles set out in the WMA Declaration of Helsinki and the Department of Health and Human Services Belmont Report. | Not Applicable | |
| Studies involving **human participants:** For publication of **patient photos**, include a statement confirming that consent to publish was obtained. | Not Applicable | |
| Studies involving experimental **animals**: State details of **authority granting ethics approval** (IRB or equivalent committee(s), provide reference number for approval. Include a statement of compliance with ethical regulations. | Yes | Material and Methods |
| Studies involving **specimen and field samples:** State if relevant **permits** obtained, provide details of authority approving study; if none were required, explain why. | Not Applicable | |

| **Dual Use Research of Concern (DURC)** | **Information included in the manuscript?** | **In which section is the information available?** (Reagents and Tools Table, Materials and Methods, Figures, Data Availability Section) |
|---|---|---|
| Could your study fall under dual use research restrictions? Please check biosecurity documents and list of **select agents and toxins** (CDC): https://www.selectagents.gov/sat/list.htm | Not Applicable | |
| If you used a select agent, is the security level of the lab appropriate and reported in the manuscript? | Not Applicable | |
| If a study is subject to dual use research of concern regulations, is the name of the **authority granting approval and reference number** for the regulatory approval provided in the manuscript? | Not Applicable | |

## Reporting

The MDAR framework recommends adoption of discipline-specific guidelines, established and endorsed through community initiatives. Journals have their own policy about requiring specific guidelines and recommendations to complement MDAR.

| **Adherence to community standards** | **Information included in the manuscript?** | **In which section is the information available?** (Reagents and Tools Table, Materials and Methods, Figures, Data Availability Section) |
|---|---|---|
| State if relevant guidelines or checklists (e.g., **ICMJE, MIBBI, ARRIVE, PRISMA**) have been followed or provided. | Not Applicable | |
| For **tumor marker prognostic studies,** we recommend that you follow the **REMARK** reporting guidelines (see link list at top right). See author guidelines, under 'Reporting Guidelines'. Please confirm you have followed these guidelines. | Not Applicable | |
| For **phase II and III randomized controlled trials**, please refer to the **CONSORT** flow diagram (see link list at top right) and submit the CONSORT checklist (see link list at top right) with your submission. See author guidelines, under 'Reporting Guidelines'. Please confirm you have submitted this list. | Not Applicable | |

## Data Availability

| **Data availability** | **Information included in the manuscript?** | **In which section is the information available?** (Reagents and Tools Table, Materials and Methods, Figures, Data Availability Section) |
|---|---|---|
| Have **primary datasets** been deposited according to the journal's guidelines (see 'Data Deposition' section) and the respective accession numbers provided in the Data Availability Section? | Not Applicable | Datasets included in supplementary data 1, 2 and 3 |
| Were **human clinical and genomic datasets** deposited in a public access-controlled repository in accordance to ethical obligations to the patients and to the applicable consent agreement? | Not Applicable | |
| Are **computational models** that are central and integral to a study available without restrictions in a machine-readable form? Were the relevant accession numbers or links provided? | Not Applicable | |
| If publicly available data were reused, provide the respective **data citations in the reference list**. | Not Applicable | |
